# Ultrafast photogeneration of a metal–organic nitrene from 1,1′-diazidoferrocene†

**DOI:** 10.1039/d4sc00883a

**Published:** 2024-04-09

**Authors:** Frederik Scherz‡, Markus Bauer‡, Luis I. Domenianni, Carolin Hoyer, Jonas Schmidt, Biprajit Sarkar, Peter Vöhringer, Vera Krewald

**Affiliations:** a Department of Chemistry, Theoretical Chemistry, TU Darmstadt Peter-Grünberg-Str. 4 64287 Darmstadt Germany vera.krewald@tu-darmstadt.de; b Clausius-Institut für Physikalische und Theoretische Chemie, Rheinische Friedrich-Wilhelms-Universität Bonn Wegelerstraße 12 53115 Bonn Germany p.voehringer@uni-bonn.de; c Institute of Inorganic Chemistry, University of Stuttgart Pfaffenwaldring 55 70569 Stuttgart Germany biprajit.sarkar@iac.uni-stuttgart.de; d Institut für Chemie und Biochemie, Freie Universität Berlin Fabeckstraße 34-36 14195 Berlin Germany

## Abstract

Ferrocene and its derivatives have fascinated chemists for more than 70 years, not least due to the analogies with the properties of benzene. Despite these similarities, the obvious difference between benzene and ferrocene is the presence of an iron ion and hence the availability of d-orbitals for properties and reactivity. Phenylnitrene with its rich photochemistry can be considered an analogue of nitrenoferrocene. As with most organic and inorganic nitrenes, nitrenoferrocene can be obtained by irradiating the azide precursor. We study the photophysical and photochemical processes of dinitrogen release from 1,1′-diazidoferrocene to form 1-azido-1′-nitrenoferrocene with UV-pump–mid-IR-probe transient absorption spectroscopy and time-dependent density functional theory calculations including spin–orbit coupling. An intermediate with a bent azide moiety is identified that is pre-organised for dinitrogen release *via* a low-lying transition state. The photochemical decay paths on the singlet and triplet surfaces including the importance of spin–orbit coupling are discussed. We compare our findings with the processes discussed for photochemical dinitrogen activation and highlight implications for the photochemistry of azides more generally.

## Abstract 2

A bent azide intermediate is key to form 1-azido-1′-nitrenoferrocene from 1,1′-diazidoferrocene, as shown with UV-pump–mid-IR-probe transient absorption spectroscopy and density functional theoryTD-DFT calculations including spin–orbit coupling.

## Graphical abstract

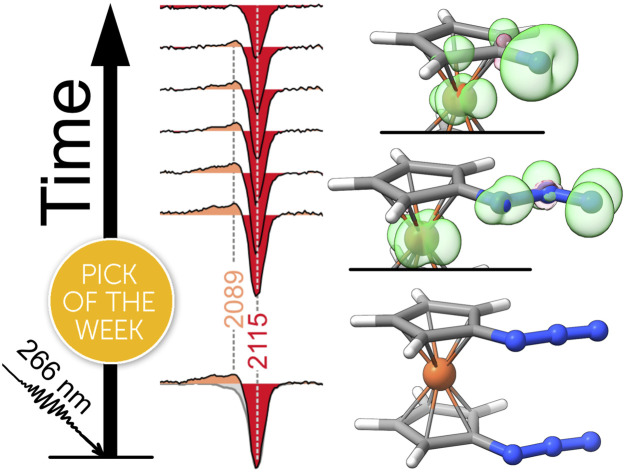

## Introduction

Ferrocene is an iconic molecule, the discovery of which has revolutionized our ideas regarding organometallic compounds and chemical bonding in molecules.^1,2^ Ever since its discovery and structural elucidation,^3–6^ discussions on the properties of ferrocene, particularly its substitution reactions, have been dominated by its analogy with another famous molecule, benzene.^7^ In fact, the name ferrocene itself was coined by its analogy with benzene, as both molecules were deemed to display similar reactivity in electrophilic aromatic substitution reactions. Despite these similarities, the presence of the iron ion in ferrocene constitutes a fundamental difference to benzene: the availability of d-orbitals can play a decisive role in its properties and reactivity. While the iron ion and its d-orbitals have always been considered important for properties, *e.g.*, its redox chemistry, its role in shaping the reactivity of ferrocene is perhaps underappreciated.

Despite having been known for more than 70 years,^8^ ferrocene and its derivatives continue to fascinate chemists. This is evidenced in recent reports, for instance on high-valent and low-valent ferrocene containing compounds,^9–11^ and the use of ferrocene containing compounds as redox-switches in catalysis.^12–19^ Given the fascination with ferrocene, its important chemistry and its continued analogies with benzene, we set out to investigate the photochemistry of 1,1′-diazidoferrocene, which yields a nitrene species, 1-azido-1′-nitrenoferrocene.

Aromatic nitrenes^20^ are highly elusive molecules that can form as the primary products of the photolysis of organic azides; that is, through N_α_–N_β_ bond cleavage with elimination of a dinitrogen molecule:R–N=N^+^=N^−^ + *hν* → R–N + N≡N

Just like their carbene congeners, they can have singlet or triplet electronic configurations; more specifically, a triplet (^3^A_2_), an open-shell singlet (^1^A_2_), and two closed-shell singlet (^1^A_1_) electronic configurations. Aromatic nitrenes have found a number of intriguing applications in the life and materials sciences ranging from photoaffinity labeling for studying structure–function relationships in biochemistry to photochemical crosslinking of polymeric systems for lithographic purposes in microelectronics.^21–24^ In liquid solution and in the presence of trapping agents, photogenerated aromatic nitrenes continue to react to a variety of secondary products, including the dimerized azo species, R–N=N–R, and eventually some polymeric tars of complex composition.^25–29^

A well-known organic nitrene is phenylnitrene, Ph–N, generated by photolysis of phenylazide, Ph–N_3_. Sophisticated matrix-isolation spectroscopy identified secondary products of Ph–N_3_ such as benzazirine and its ring-expanded isomer, a cyclic keteneimine.^30–35^ The rich photochemistry involving aromatic nitrenes has also been explored in liquid solution using flash photolysis and time-resolved spectroscopy^36–44^ and has been reviewed comprehensively in the literature.^45,46^

**Fig. 1:**
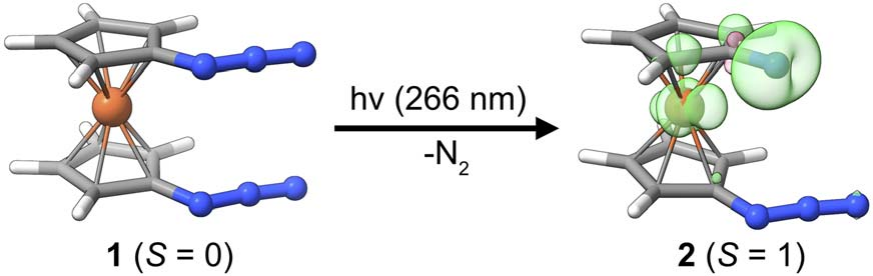
Molecular structures of Fc(N_3_)_2_ (*S* = 0) and Fc(N_3_)(N) (*S* = 1) from DFT calculations. The spin density of the product is distributed over the nitrene-N-atom and the iron ion (*cf.* green and pink 3D-contours with a contour value of 0.01. Loewdin spin populations, Fe: 0.6, N: 1.1).

Herein, we present the photochemical generation of 1-azido-1′-nitrenoferrocene from 1,1′-diazidoferrocene, see Fig. 1, and discuss the involvement of iron d-orbitals and spin–orbit coupling on this process. We can thus contrast the photochemistry of this metal–organic azide with that of the well-established phenylazide and address the fundamental question whether the photochemistry of azido-ferrocenes can be considered an extension of phenylazide photochemistry. The photochemical path for 1-azido-1′-nitrenoferrocene generation is elucidated with UV-pump–mid-IR-probe transient absorption spectroscopy and time-dependent density functional theory calculations considering spin–orbit coupling. We identify an intermediate with a bent azide moiety that is preorganized for dinitrogen release *via* a small thermal barrier. The electronic structure of this intermediate and implications for other photochemical azide splitting processes are discussed. Finally, we draw some analogies to the processes recently discussed in the context of dinitrogen photoactivation.

## Results and discussion

### Structures and electronic structures

To explore the possibility of a photoinduced conversion of a metal–organic azide to the respective nitrene, we studied the closed-shell (*S* = 0) precursor complex, 1,1′-diazidoferrocene (Fc(N_3_)_2_ or [N_3_–Cp⋯Fe⋯Cp–N_3_], Fig. 1). We monitored its photoinduced primary processes with femtosecond ultraviolet-pump/mid-infrared-probe (UV/MIR) spectroscopy in conjunction with DFT computations. The nitrene photoproduct expected to form upon loss of one dinitrogen molecule according to[N_3_–Cp⋯Fe⋯Cp–N_3_] + *hν* → [N_3_–Cp⋯Fe⋯Cp–N] + N_2_is 1-azido-1′-nitrenoferrocene, Fc(N_3_)(N) or [N_3_–Cp⋯Fe⋯Cp–N]. By choosing the diazido species rather than Fc(N_3_), the product retains an N_3_ moiety, which serves as a suitable infrared chromophore for the nascent nitrene.

The parent complex 1 is a singlet species with the triplet predicted at +66.2 kJ mol^−1^ by DFT calculations (D4-PBE0/def2-TZVP, CPCM(MeCN), see Computational details in the ESI†). It dominantly adopts a fully eclipsed conformation, in agreement with the crystal structure.^47^ For both the *syn* and the *anti* conformers of the azides, the barrier for mutual rotation of the Cp–N_3_ units is below 10 kJ mol^−1^ (see ESI, Table S6†). Therefore, properties are calculated as Boltzmann-weighted averages of the properties of five *syn* and five *anti* rotamers. While isolated Cp–nitrene has a singlet ground state, the expected ferrocenyl nitrene product 2 is predicted to have a triplet ground state (*S* = 1) with the spin density distributed over the nitrene nitrogen atom and the central iron ion; see Fig. 1. Notably, phenylnitrene has a triplet ground state,^48,49^ highlighting the analogy to the ferrocenyl nitrene discussed here. For the putative photoproduct, the rotational barrier is even lower (*ca.* 4 kJ mol^−1^, see Table S10†) than for the diazido parent compound so that the same Boltzmann-weighting approach for the prediction of properties is used.

### UV-pump/MIR-probe spectral evolution

**Fig. 2:**
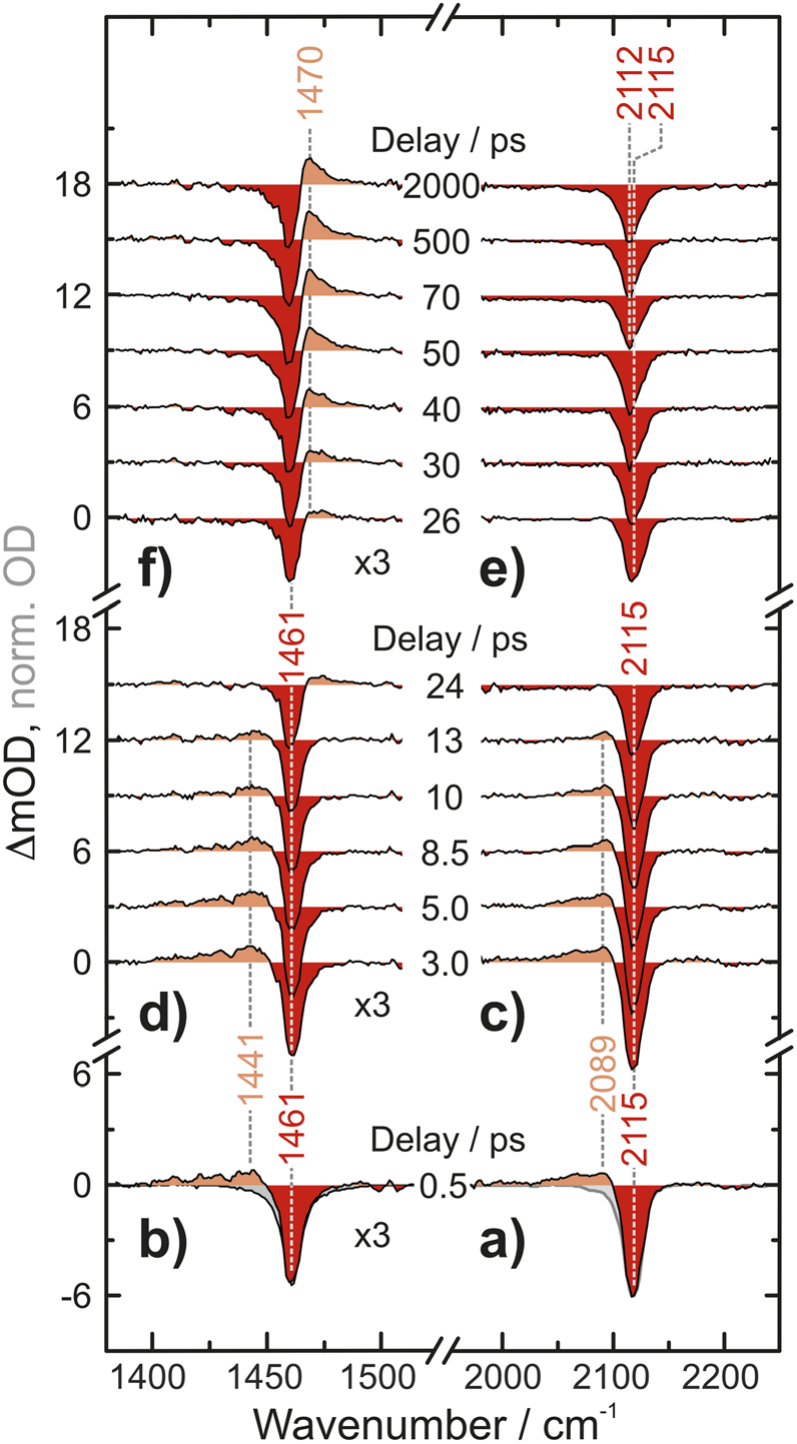
UV-pump–MIR-probe spectrum recorded in deuterated acetonitrile at a delay of 500 fs in (a) the N_3_ antisymmetric stretching region and (b) the CN/CH-region. (c and d) Spectral evolution on a time scale of up to 24 ps. (e and f) Spectral evolution on a time scale of up to 2000 ps. The grey areas in (a and b) correspond to inverted and scaled down absorptions of the parent compound.

UV-pump/MIR-probe spectra of Fc(N_3_)_2_ in liquid acetonitrile are shown in Fig. 2 for various time delays ranging between 0.5 ps and 2 ns after irradiation at 266 nm. At a very short delay of 0.5 ps (Fig. 2a and b), the distinct negative features peaking at 1461 cm^−1^ and 2115 cm^−1^ represent ground state bleaches (GSB), which arise directly from the absorption of the pump photon and the concomitant population depletion of the electronic ground state of the diazide parent. In addition, pronounced positive features (*i.e.*, induced absorptions) are detected on the low-frequency edge of the each GSB (in the N_3_-antisymmetric stretching region at 2089 cm^−1^ and in the CN/CH-region at 1441 cm^−1^). Even at this very short delay, the frequency-integrated absorption-to-bleach ratio in the azide region is *ca.* 1/2 suggesting that one of the two N_3_-moieties of the parent complex has already lost its prototypical antisymmetric stretching character.

In the experimental data recorded at intermediate delays of up to 13 ps, Fig. 2c and d, the two induced absorptions decay simultaneously while the GSBs partially recover. On even longer time scales of up to 2000 ps, Fig. 2e and f, a second induced absorption gradually builds up in the CN/CH-region. The maximum is found at around 1470 cm^−1^, *i.e.*, now blueshifted with respect to the corresponding GSB. Concurrently, a subtle redshift of *ca.* 3 cm^−1^ of the azide GSB is observed. After 2000 ps, the negative signal reaches an asymptotic position of 2112 cm^−1^ suggesting that the bleach is partially obscured on its high-frequency edge.

### Assignment of UV-vis spectra and photochemical path

**Fig. 3:**
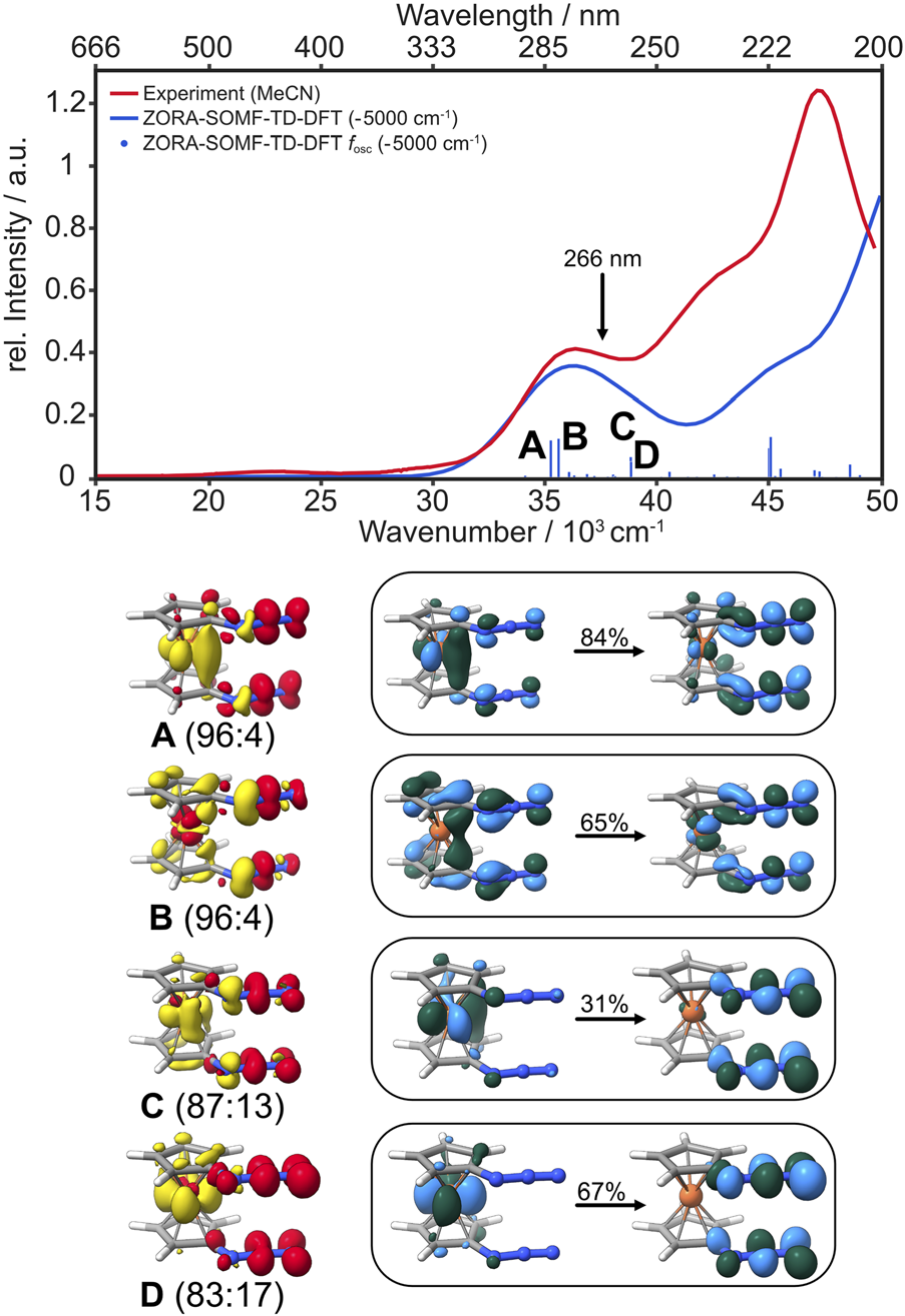
Experimental UV/vis absorption spectrum of Fc(N_3_)_2_ in liquid acetonitrile solution (red) and the TD-DFT computed spectrum considering spin–orbit coupling between singlet and triplet states (blue; ZORA-D4-CAM-B3LYP/def2-TZVP//D4-PBE/def2-TZVP incl. SOMF). The characters of the vertical transitions labeled A, B, C, D in the spectrum are represented as difference densities (yellow: density loss; red: density gain, contour value 0.0025) and natural transition orbitals (NTOs; light blue/green, contour value 0.05) of the dominant singlet components (see Fig. S4–S11† for the minority components and triplet difference densities).

The electronic absorption spectrum of 1,1′-diazidoferrocene has two well separated, broad bands at 447 nm and 275 nm, and a higher energy band at 210 nm with a shoulder at 233 nm, see Fig. 3. Irradiation at 266 nm induces the release of dinitrogen.

TD-DFT calculations (CAM-B3LYP/def2-TZVP) considering spin–orbit coupling between singlet and triplet states in each conformer are in good agreement with experiment. The low-intensity band at 447 nm arises from d–d transitions. The band at 275 nm contains charge transfer (CT) transitions into azide-centred LUMOs of π*π* character. The intense transitions A–D have small to moderate degrees of spin–orbit coupling (S : T in A: 96 : 4, in B: 96 : 4, in C: 87 : 13, in D: 83 : 17) and are predominantly metal-to-ligand charge transfer states. All acceptor orbitals in A–D are azide π*π* orbitals; the donor orbitals in A, C and D are metal-centred whereas in B they have Cp and azide π character, see NTOs in Fig. 3. We note that in the more strongly spin–orbit coupled states, the acceptor orbitals are oriented in-plane, *i.e.*, not conjugated with the aromatic system of the Cp ring.

To elucidate the photochemical path, a relaxed surface scan along the N_α_–N_β_ distance was carried out, showing that the bright states A and B are not associated with dinitrogen release along this coordinate, see Fig. S13† (*cf.* Fig. S14,† cyan trace). The excited state surface of A has a minimum at 1.28 Å (*cf.* 1.22 Å in the ground state) that is *ca.* 4 kJ mol^−1^ lower in energy than the vertical excitation. Spin–orbit coupling increases significantly along the scan and peaks at 1.28 Å with a singlet : triplet ratio of 56 : 43. Following the state characters, two essentially dissociative surfaces can be identified: a spin-pure singlet and a spin-pure triplet state, see Fig. S14,† green traces. This suggests that rapid internal conversion and intersystem crossing processes can populate these lower-lying dark states with extremely flat profiles. Notably, the minimum energy of the triplet state is lower than that of the singlet state (Δ*E*(T,S) = 31 kJ mol; see ESI Fig. S13,† and *cf.* the discussion by Budyka^50,51^), but both states run in parallel over the scan range and have a similar barrier of 8–10 kJ mol^−1^ for dinitrogen release.

Two very similar states appear in a relaxed surface scan of phenylazide with remarkable correspondence to the ferrocene case in terms of energy and evolution (*cf.* Fig. S14 and S16†). In contrast, a scan of the cyclopentadienylazide anion shows different behaviour (Fig. S18†), implying that azides in general have a wider variance in electronic structure evolution along the N–N_2_ dissociation path. This finding provides another example for the longstanding analogies between ferrocene and benzene.

Considering again all spatial degrees of freedom for 1,1′-diazidoferrocene, a potential candidate for the spectroscopically emerging species was identified by wavefunction overlap-based excited state geometry optimization of several excited states,^52^ resulting in the distinct bending of one azide moiety. An intermediate with one bent azide was identified as a minimum on the triplet surface. In contrast, structural relaxation of this species on the singlet surface, either as an open-shell or a closed-shell singlet, results in a return to the ground state structure with two linear azides.

The key structural features of the intermediate are a significantly diminished N_α_–N_β_–N_γ_ bond angle (126° *vs.* 174° in the ground state) and an elongated N_α_–N_β_ bond (1.38 Å *vs.* 1.22 Å in the ground state). This can be rationalized by the N_β_ atom changing from sp-hybridization in the ground state to sp^2^-hybridization in the triplet intermediate and a concomitantly weakened bond to the N_α_ atom. The spin density of the triplet intermediate indicates population of a metal-centred orbital and an in-plane azide π*-orbital. Notably, there is positive spin density along the N_α_–N_β_ axis. As discussed further below, SOC enables population of the π-system parallel to the Cp plane which leads to a torque on the distal N_2_ unit.

**Fig. 4:**
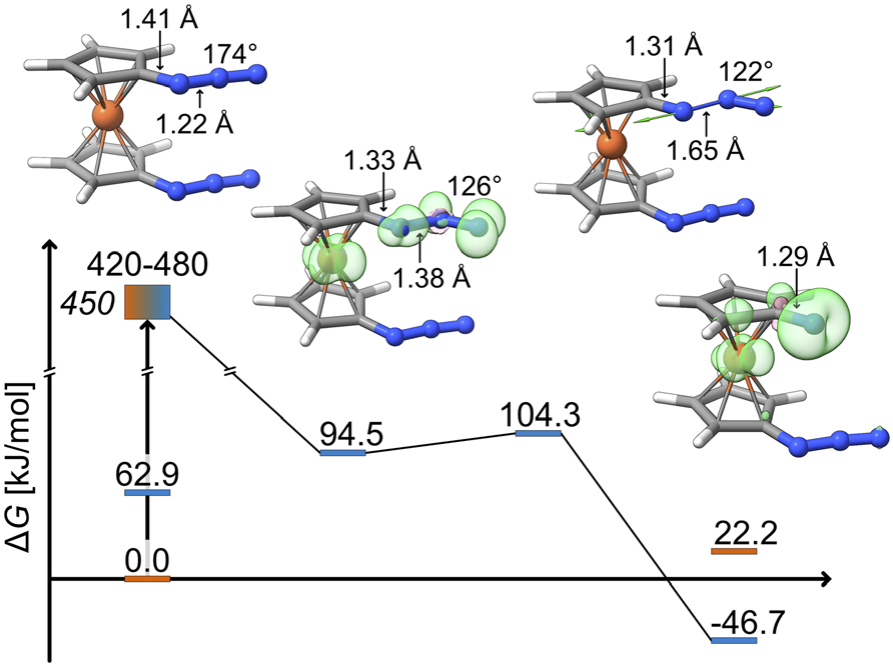
Gibbs free energy profile in kJ mol^−1^ for the photolytic N_2_ dissociation pathway proposed by DFT (D4-PBE0/def2-TZVP). The experimental irradiation energy is given in italics. Loewdin spin populations for the intermediate, Fe: 1.1, (N_α_, N_β_, N_γ_): (0.2, 0.2, 0.6), and product, Fe: 0.6, N: 1.1.

The bent nature of the azide in this intermediate suggests that it is pre-organized for dinitrogen release. Indeed, a low-lying transition state (Δ*G*_a_ = 9.8 kJ mol^−1^, see Fig. 4) is found for the exergonic dissociation of a closed-shell N_2_ molecule and formation of the triplet ferrocenylazide nitrene product (Δ*G* = −46.7 kJ mol^−1^).

**Fig. 5:**
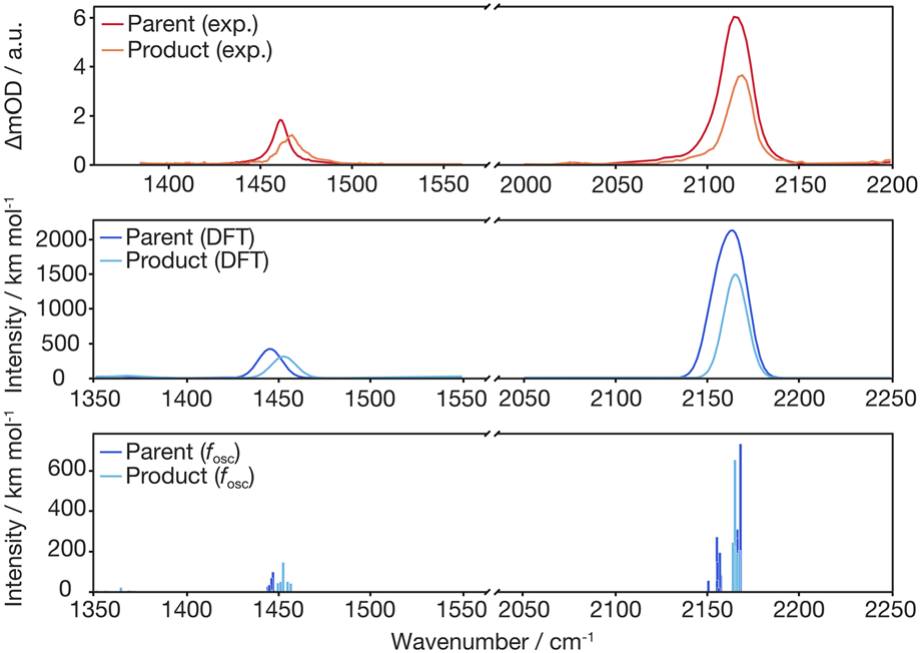
Infrared spectra of the parent Fc(N_3_)_2_ (FTIR) and the photoproduct (data analysis at a 2000 ps pump–probe delay) in liquid d_3_-acetonitrile (for *ν̃* ≤ 1700 cm^−1^) and dichloromethane (for *ν̃* > 1700 cm^−1^) solution at room temperature (top) compared to the DFT computed, Boltzmann-weighted IR bands of conformers of the Fc(N_3_)_2_ ground state and the nitrene species at 298 K (middle: individual transitions broadened with FWHM of 15 cm^−1^; bottom: dominant individual contributions of stacked azide conformers, see ESI† for details).

With this proposal for the photochemical path in hand, the putative intermediate and the suggested nature of the product can be connected to the experimental data. The IR bands of the two azides serve as spectroscopic probes of the excited state decay. The experimental data were collected in dichloromethane (transparent window for the azide stretching region: 1700–2200 cm^−1^) and fully deuterated acetonitrile (transparent window for the C–N stretching region: 1300–1700 cm^−1^), see Fig. 5. The IR-absorption of the azide antisymmetric stretching vibration of diazidoferrocene is found at 2115 cm^−1^ (comp.: 2163 cm^−1^ for the Boltzmann-weighted average). There are two such vibrations in each conformer; their intensities and energetic splittings depend on the relative azide orientations. This is expected as for an idealized collinear alignment of two azides, the dipole moment of the out-of-phase normal mode will vanish whereas that of the in-phase component is maximal. The C–N stretching absorption is found at 1461 cm^−1^. The computations show an in-phase C_1/1′_–N_α/α′_-stretching normal mode mixed with a C–H in-plane bending motion of the two Cp-rings at 1445 cm^−1^ for the Boltzmann-weighted average (see ESI, Fig. S22†).

To obtain an experimental IR spectrum of the pure product, the properly scaled stationary FTIR spectrum of the parent was added to the raw pump–probe data recorded after 1 ns (*vide infra*), thereby removing all contaminating negative ground state bleach signals. The product bands in both regions of interest are blueshifted relative to those of the parent obscuring the respective high frequency edges of the GSBs, see Fig. 5. The predicted IR bands for a Boltzmann-weighted average of the anticipated triplet nitrene product conformers show excellent agreement in the relative band positions and intensity ratios. This suggests that the dominant species after 1 ns is indeed the triplet 1-azido-1′-nitrenoferrocene product, [N_3_–Cp⋯Fe⋯Cp–N], and defines a steady state, or an endpoint of the photoinduced primary events.

Having now assigned the product spectrum to the nitrene species, we address the question of when the product forms. We note that the scaled parent spectrum required to remove the GSB contribution from the 500 fs-UV/MIR-data corresponds to the total absorption of those molecules that have been converted photolytically by the pump pulse. Therefore, it is permissible to directly compare the amplitudes of the two sets of experimental spectra displayed in Fig. 5. In the CN/CH-region, the ratio between the frequency-integrated absorptions of product and parent is exactly equal to one, whereas in the azide region, it is equal to 1/2. This finding is highly suggestive of a primary quantum yield for nitrene formation of 100%. The frequency-integrated absorption-to-bleach ratio in the azide stretching region is *ca.* 1/2 already at a time delay of 0.5 ps. We can therefore conclude that one of the two N_3_-moieties of the parent complex has lost its prototypical antisymmetric stretching character already at 0.5 ps, which would be consistent with formation of the intermediate with the bent azide moiety or even a complete loss of an N_2_-fragment. The sensitivity of the azide stretching frequency to this structural distortion is elucidated with a ground state scan of the azide bending angle (see ESI, Fig. S21†). Performing this scan in the ground state is a reasonable approximation for the expected qualitative changes in the excited state(s) that are not directly accessible. Bending the N_α_–N_β_–N_γ_ moiety by 10° leads to a red-shift of 36 cm^−1^ at an energetic cost of only *ca.* 10 kJ mol^−1^. This points towards the experimentally observed redshift originating from the intermediate with the bent azide moiety.

### Kinetics

**Fig. 6:**
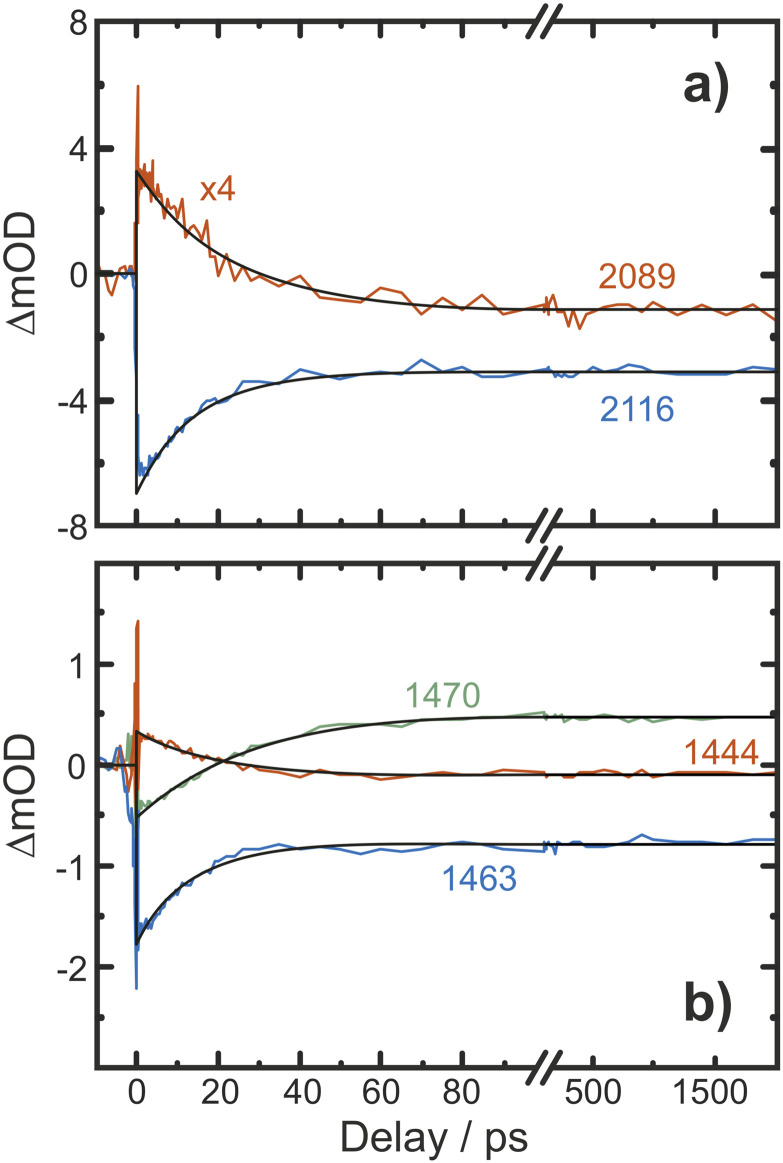
Pump–probe kinetic traces recorded in (a) the azide antisymmetric-stretch region and (b) in the CN/CH-region. The black curves represent fits of the transients to single-exponential kinetics.

**Fig. 7:**
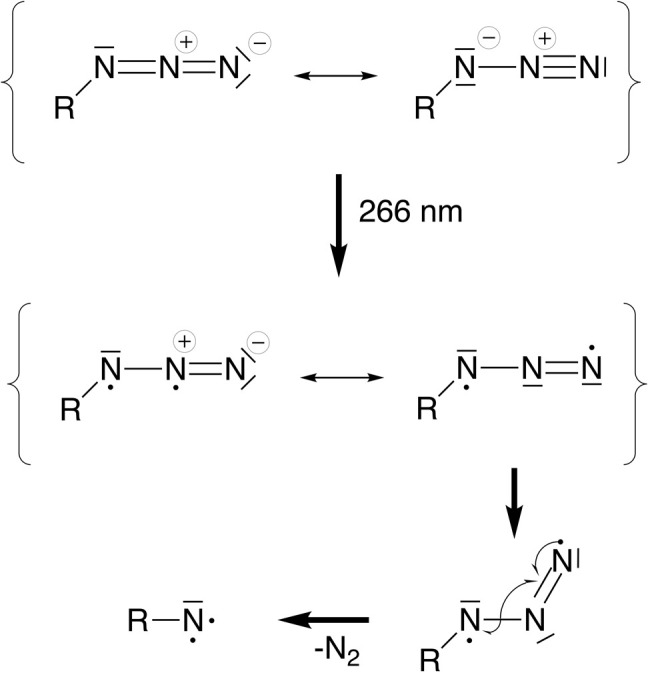
Valence bond interpretation of the electronic and geometric structure changes after excitation into π*π* orbitals. Upon photoexcitation, a diradical species is generated with at least one unpaired electron being delocalized over the π system of the azide moiety. One mesomeric structure (middle right) shows that the central nitrogen atom is now formally sp^2^-hybridized, rationalizing the geometric change from a linear (*ca.* 180°) to a bent (*ca.* 120°) geometry of the azide unit. In the bent geometry, the in-plane π system is no longer orthogonal to the R–N σ bond, allowing them to interact to release N_2_ and generating the nitrene.

Finally, we inspect representative pump–probe time traces to obtain information regarding the kinetics of formation of the final photochemical product, Fig. 6. In the CN/CH region, the initially downshifted induced absorption peaking at 1441 cm^−1^ decays on a time scale of several tens of picoseconds. A kinetic trace recorded at 1470 cm^−1^ displays an initial ground state bleach, which subsequently changes sign on the very same time scales, thereby signaling the build-up of the upshifted secondary absorption peaking at 1465 cm^−1^. Fitting these two traces to single-exponential kinetics to which a constant offset is added yields nearly identical time constants of 16 ps and 18 ps, respectively. This indicates that the species giving rise to the early downshifted absorption is the precursor of the final photochemical product observed after 200 ps at 1470 cm^−1^. The apparent recovery of the bleach recorded at 1463 cm^−1^ (blue trace in Fig. 7b) can then be understood as a superposition of the kinetics of these two species and a prompt, permanent parent ground state bleach. In the azide region (Fig. 6a), the initial downshifted absorption around 2089 cm^−1^ also decays in a single-exponential fashion with a time constant of 19 ps, which agrees very well with that obtained from the CN/CH-region. Likewise, the azide ground-state bleach recovers mono-exponentially with a time constant of 22 ps, again in good agreement with the absorption decay time. Once again, the apparent bleach recovery dynamics in this region do not result from a time-dependent recovery of population of the parent's electronic ground state but rather from the dynamic growth of the nitrene product absorption.

Overall, the UV/MIR spectro-temporal evolution in combination with our computational studies provide compelling evidence for the “prompt” loss (within our time-resolution) of a single azide absorber, which we attribute to the formation of an excited-state intermediate featuring a bent-N_3_ moiety. According to theory, the asymmetric azide stretching absorption profile at a delay of 500 fs results from (i) the intermediate occupying a potential energy well that is rather shallow along the N_3_-bending coordinate, and (ii) motion along the bending coordinate coupling strongly to the azide antisymmetric stretching vibration (*i.e.* the off-diagonal N_3_-stretch-bend anharmonic coupling). In addition, vibrational excitation of low frequency modes may also contribute to the broad line shape.

Having identified not only the intermediate but also the transition state (TS) for its loss of dinitrogen, a rate constant, *k*, for nitrene formation can be estimated using the classical expression*k* = *cν̃*_0_e^−Δ*E*/*k*_B_*T*^where Δ*E* is the classical barrier height. The quantity, *cν̃*_0_, represents a maximal rate (or equivalently, a barrier crossing attempt frequency), which can be estimated from the frequency of the local N_α_–N_β_ stretching motion of the bent intermediate. Decomposing its normal modes into the local mode basis using the LModeA package from Kraka and coworkers yields a N_α_–N_β_ stretching wavenumber of 859 cm^−1^.^53^ Together with the electronic energy difference Δ*E* = 17.1 kJ mol^−1^ (Table S14†) from above, we arrive at a rate constant for N_2_ loss of 1/(38 ps), which is on the same order of magnitude as the experimentally observed rate of 1/(12 ps).

Alternatively, canonical transition state theory can be used,
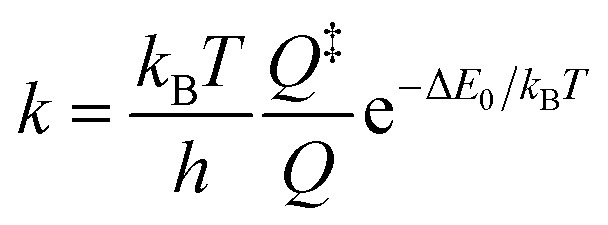
where *k*_B_ is Boltzmann's constant, *T* is the temperature, and *h* is Planck's constant. The quantities *Q* and *Q*^‡^ represent the vibrational partition functions of the bent intermediate and the TS, and are both referenced to the respective zero-point energy. Taking the zero-point energies into account, the barrier height, Δ*E*_0_, for N_α_–N_β_ cleavage amounts to 10.9 kJ mol^−1^ (Δ*E* + ΔZPE, see Table S14†). The calculation yields a rate constant of 1/(7 ps), which is less than a factor of two larger than the experimentally observed rate of 1/(12 ps).

## Discussion

Bending of a linear unit by excitation into the π system and hence opening a spatial degree of freedom may be relevant to light-induced bond splitting reactions more generally. In the context of dinitrogen photoactivation,^54,55^ this was deduced from transient absorption spectroscopy studies, albeit without the observation of intermediates. In two complexes, (μ-N_2_)[Mo(N[^*t*^Bu]Ar)_3_]_2_ with Ar = 3,5-C_6_H_3_Me_2_ (ref. 56) and (μ-N_2_)[W(CO)(^t^BuPNP)]_2_,^57^ electron–hole recombination was seen on a sub-ps timescale, leading to the conclusion that N–N dissociation resulted from a vibrationally hot ground state where the previously linear core might be bent in zig-zag fashion. In both cases, a non-statistical vibrational energy distribution was deduced.^56,57^ The same had already been observed in the photochemistry of *trans*-[(cyclam)Fe^III^(N_3_)_2_]^+^.^58^

Our findings are furthermore consistent with literature suggestions of bent azide species.^59–67^ To deduce guiding principles for interpreting or even designing photochemistry, the questions of preferred geometric structure and ideal electronic structure for a desired product, such as a nitrene, must be linked.

In a simplified molecular orbital picture, excitation into an azide π*π* orbital weakens the double bond and opens an additional degree of freedom that allows bending of the azide unit. Population of the triplet state *via* ISC implies a torque on the N_2_ fragment since the π*π* orbital orthogonal to the π-system of the Cp ring is populated. In a valence-bond picture, Fig. 7, the excited state may be written as a diradical with two adjacent formal charges or with formally neutral nitrogen atoms which is energetically preferred. In a triplet electronic configuration, the unpaired electrons must be located in orthogonal orbitals, whereas in a singlet configuration the unpaired electrons can occupy the same physical space which may lead to rapid relaxation back into the ground state. The triplet configuration thus appears beneficial for dinitrogen release since it prevents recombination of the unpaired electrons and allows sufficient time for forming the bent azide intermediate.

With these arguments and the calculations available for the present case, the triplet surface appears as the more relevant one. However, with a more general view on azide photochemistry, the singlet surface should not be overlooked. In the present case, both multiplicities have minima in a scan of the N_α_–N_β_ distance (Δ*E*(T,S) = 31 kJ mol^−1^, triplet more stable), and it is plausible that this will be the case in any azide. Returning to the comparison of 1,1′-diazidoferrocene and phenylazide, the singlet and triplet states run in parallel in the N_α_–N_β_ scans with near-identical structures and energies. A notable difference is the higher degree of SOC in the iron compound, which should make the triplet surface more easily accessible. In accordance with the Pauli exclusion principle, geometry optimization of the singlet diazidoferrocene leads back to the singlet ground state structure, whereas the intermediate with the triplet electronic structure is prevented from relaxing back into the ground state if spin–orbit coupling is significantly diminished. This view may suggest that only the triplet surface leads to the triplet product. However, opportunities for ISC energetically above the triplet surface and spatially outside the region explored in N_α_–N_β_ distance scans cannot be excluded for azides in general, and thus a reaction path that involves the singlet minimum with a later crossing onto the triplet surface can in principle exist. Platz and coworkers suggested phenylazide photolysis to involve independent singlet and triplet product channels, whereas computationally, ISC was proposed to precede N_2_ release.^68^ For organic azides, and phenyl azide in particular, Budyka stated that N_2_ release occurs in the excited singlet azide.^40,50,51,69,70^ Clearly, it will depend on the conformational flexibility and electronic structure of a specific azide, including the energetic gap and the degree of SOC between the singlet and triplet surfaces, which states are traversed during the photophysical and photochemical relaxation paths.

From a design perspective, the singlet surface appears *a priori* less productive, and perhaps even dangerous if the desired outcome is the photocleaved product: the decay channel back into the ground state is not spin-forbidden and hence never fully precluded. A promising rationale for the design of azide photochemistry might thus be to stabilize the triplet minimum of a bent azide structure, so that a significant population can accumulate there and cool vibrationally. Assuming minimal SOC, this may either enable (to date unknown) excited state reactivity of the bent azide species in the triplet state, or, depending on the barrier height, push the system into the N_2_-release channel to yield the triplet nitrene species. The latter idea was realized in a recent study of the photochemistry of square-planar [Pt(PNP)(N_3_)] with a singlet ground state, where the N_2_-releasing intermediate was identified as the lowest triplet state with azide ππ* character.^64^ This state has a bent azide moiety and facilitates N_2_ ejection on a near-barrierless, adiabatic triplet surface.

## Conclusion

To summarize, dinitrogen is expelled from Fc(N_3_)_2_ upon excitation at 266 nm to form the triplet 1-azido-1′-nitrenoferrocene product. Experimental UV-pump–MIR-probe data show a prompt induced absorption with a halved amplitude relative to that of the ground state bleach which is indicative of the loss of a prototypical azide anti-symmetric stretching absorber within the first 0.5 ps. This primary photoproduct subsequently relaxes on a time scale of *ca.* 20 ps to a secondary product, whose CN/CH- and azide-absorptions are frequency-upshifted relative to that of the parent. The relative intensities and energies of the parent and product IR signatures are in excellent agreement with the predicted modes using a Boltzmann-average of the five *syn* and five *anti* Cp/Cp′ rotamers. TD-DFT calculations considering SOC show that the photodissociation sequence is initiated by a CT excitation into azide-centered orbitals of π*π* character. Excited state geometry optimizations identify a triplet intermediate structure in which one of the two azide moieties has a bent conformation (*cf.*Fig. 2). We assign this structure to the primary photoproduct, which is prepared for dinitrogen release *via* a low-lying transition state that leads to the final photoproduct, the triplet ferrocenyl nitrene.

## Data availability

Data is available on reasonable request.

## Author contributions

FS: data collection computations, data analysis (equal), preparation of figures, contribution to writing. MB: data collection spectroscopy (equal), data analysis (equal). LID: data collection spectroscopy (equal), data analysis (equal). CH: Synthesis and ground state characterization of the compound. JS: data collection spectroscopy (equal). BS: conception (equal), design (equal), analysis (equal), writing (equal), funding (equal), supervision of CH. PV: conception (equal), design (equal), analysis (equal), writing (equal), funding (equal), supervision of MB, LID, JS. VK: conception (equal), design (equal), analysis (equal), writing (equal), funding (equal), supervision of FS.

## Conflicts of interest

There are no conflicts of interest to declare.

## Supplementary Material

SC-015-D4SC00883A-s001
