# Supplementary material for: Ultrafast photogeneration of a metal–organic nitrene from 1,1′-diazidoferrocene
Source: Chem Sci. 2024 Apr 9;15(18):6707–15. doi: 10.1039/d4sc00883a (PMC11077559; doi:10.1039/d4sc00883a)
Supplement: SC-015-D4SC00883A-s001 [file SC-015-D4SC00883A-s001.pdf]

Electronic Supplementary Material (ESI) for Chemical Science.
This journal is © The Royal Society of Chemistry 2024

Supporting Information

for

Ultrafast photogeneration of a metal-organic nitrene from 1,1'-diazidoferrocene

Frederik Scherz,[a]# Markus Bauer,[b]# Luis I. Domenianni,[b] Carolin Hoyer,[c] Jonas Schmidt,[b] Biprajit Sarkar*,[c] Peter Vöhringer*,[b] and Vera Krewald*[a]

[a] M.Sc. F. Scherz, Prof. Dr. V. Krewald
Department of Chemistry, Quantum Chemistry
TU Darmstadt
Peter-Grünberg-Str. 4, 64287 Darmstadt (Germany)
E-mail: vera.krewald@tu-darmstadt.de

[b] M.Sc. Markus Bauer, Dr. L. I. Domenianni, M.Sc. Jonas Schmidt, Prof. Dr. P. Vöhringer
Clausius-Institut für Physikalische und Theoretische Chemie
Rheinische Friedrich-Wilhelms-Universität Bonn
Wegelerstraße 12, 53115 Bonn (Germany)
E-mail: p.voehringer@uni-bonn.de

[c] M.Sc. Carolin Hoyer, Prof. Dr. B. Sarkar
Institute of Inorganic Chemistry
University of Stuttgart
Pfaffenwaldring 55, 70569 Stuttgart (Germany)
E-mail: biprajit.sarkar@iac.uni-stuttgart.de

Table of Contents

1. **Experimental Details** 2
2. **Computational Details** 2
3. **References** 3
4. **Details on Complete Active Space Calculations** 5
5. **Difference densities and natural transition orbitals** 8
6. **Exploration of the potential energy landscape in the ground state and excited states** 12
7. **Cartesian Coordinates (*Å*) and FSPE (*Eh*) of all relevant species** 23

1. Experimental Details

Synthesis

The title compound was synthesized by following procedures published in the literature.[1]

Spectroscopy

Femtosecond UV-pump/MIR-probe measurements were carried out by means of a femtosecond laser pump-probe setup already described elsewhere.[2–4] In short, a fraction of the fundamental output of a Ti:sapphire oscillator/regenerative amplifier front-end (Newport Spectra Physics, Solstice Ace, pulse duration 60 fs, center wavelength 800 nm) is frequency tripled to generate pump pulses centered at 266 nm. To produce the probe pulses, a second fraction of the same regenerative amplifier front-end output was used to drive an optical parametric amplifier (TOPAS 800, Light Conversion). The synchronous generated signal and idler pulses were frequency-downconverted to the mIR-region via difference-frequency generation in a type-I $AgGaS_2$-crystal followed by a Ge-filter used to suppress residual signal/idler light. The probe pulses were subsequently split into detection and reference pulses of similar intensities. A pump-probe relative delay was generated sending the detection pulses four times to a Ag-coated hollow-roof retroreflector mounted to a motor driven linear translation stage (Newport DLS 325, MKS Industries). In order to eliminate signal contributions arising from molecular rotations, the relative pump-probe polarization was set to the magic angle. Both detection and reference pulses were focused into the sample and later recollimated by means of two Au-coated 90° off-axis parabola (10 cm effective focal length). The UV-beam is steered through a rotating chopper disk, that blocks every other pump pulse, into a fused silica lens (f = 400 mm) in order to reduce its diameter to ~1 mm at the sample position and subsequently spatially overlapped with the detection pulses at a 5° crossing angle. Finally, both mIR pulses were independently steered onto the two entrance slits of an imaging spectrometer (Horiba, iHR 320) equipped with a 2 x 32 pixel HgCdTe-array detector (Infrared associates) that allowed for referenced detection of the pump-induced differential absorbance.

The solution was circulated with a gear pump through a home-built flow cell comprised of two CaF_2 windows separated 100 µm from each other with a Pb-spacer.

2. Computational Details

Geometry optimizations

Calculations were performed with the *ORCA 5* software package.[5] Excited state geometry optimizations were realized with *pysisyphus*.[6]

Geometries were optimized with the PBE0 density functional and the triple-ζ basis set def2-TZVP and the def2 auxiliary basis set on all atoms.[7–9] Local minima on the potential energy surface were confirmed with a frequency calculation by having zero, while transition states were characterized by having exactly one mode with an imaginary frequency.

Default SCF convergence criteria (ΔE_{SCF} <= $1x10^{-8}$ E_h) and tight optimization criteria ('TightOpt' keyword in ORCA nomenclature, ΔE <= $1x10^{-6}$ E_h) were used. The default 'defgrid2' integration grids were used. To accelerate the computations, the resolution-of-identity approximation ('RIJ') was employed. Dispersion effects were included with Grimme's D4 dispersion correction.[10] In accordance with the experimental conditions, the conductor-like polarizable continuum model (CPCM) for acetonitrile was used.[11] Relativistic effects were treated within the zeroth-order regular approximation framework (ZORA) with appropriately recontracted ZORA-def2 basis sets as implemented in ORCA.[12]

Single Point calculations

DFT single point calculations were carried out with the hybrid density functional PBE0, using the RIJCOSX approximation in conjunction with the def2/J and def2-TZVP/C auxiliary basis sets.[13,14] All other settings remained the same as in the geometry optimization. For the reaction energy profile (ΔG values) the D4-PBE0/def2-TZVP, CPCM(MeCN) level of theory was employed throughout.

For the nitrene spin state energies, additional DFT single point energies were computed with D4-PBE0 in conjunction with the ma-def2-TZVP basis set and CPCM(MeCN) for environmental effects on the optimized geometries.[15] The broken-symmetry formalism was employed to compute the open-shell singlet states. The Loewdin spin populations of the open-shell singlet and triplet states are shown in Table S4 and Table S5.

SA-CASSCF(10,*n*)-NEVPT2/ma-def2-TZVP, CPCM(MeCN) calculations were performed with *n* = 9 for the Ph-N and *n* = 8 for the $[Cp\text{-}N]^-$. The calculations were averaged over one triplet and two singlet roots, capturing the open- and closed-shell singlet states.

Time-dependent DFT calculations

Relativistic spin-orbit mean-field TDDFT calculations were performed with the range-separated hybrid functional ZORA-D4-CAM-B3LYP/ZORA-def2-TZVP, CPCM(MeCN) and 120 roots were computed without the Tamm-Dancoff approximation.[16,17]

This computational protocol differs from the protocol employed for computing the single point energies (used in computing the ΔG values of the reaction pathway) by using a different functional for the electronic energies. Here the range-separated CAM-B3LYP functional was chosen because its simulated spectrum most closely matched the experimental UV/vis spectrum.

The SOMF-TDDFT formalism computes singlet and corresponding spin-adapted triplet excited states which are then coupled, so each SOC state is described as a composite of spin-pure states. It is possible to compute difference densities for the spin-pure states, however the corresponding natural transition orbitals (NTOs) are currently only accessible for the reference singlet states and not for the spin-adapted triplet excitations.

Magnitudes of selected spin-orbit coupling matrix elements (SOCMEs) were calculated as the square root of the sum of the squared vector elements for each SOCME. This corresponds to calculating the length of the associated spin-orbit coupling vector. For SOC states comprised of multiple pure singlet/triplet state pairs, their SOCMEs were weighted by the ratio of the state pairs making up the SOC state.

Relaxed Potential Energy Surface Scans

Geometries along the scan coordinates were generated by fixing the desired parameter of the ground state structure to a predetermined value and conducting a constrained geometry optimization at the D4-PBE/def2-SVP, CPCM(MeCN) level of theory.

The optimized geometries were used as input for SOMF TD-DFT calculations at the D4-CAM-B3LYP/def2-TZVP, CPCM(MeCN) level of theory with 32 roots and the Tamm-Dancoff approximation enabled.

Visualisation

Molecular structures and densities were rendered with the *SEQCROW* plugin for UCSF *ChimeraX*. (*UCSF ChimeraX*: developed by the Resource for Biocomputing, Visualization, and Informatics at the University of California, San Francisco, with support from National Institutes of Health R01-GM129325 and the Office of Cyber Infrastructure and Computational Biology, National Institute of Allergy and Infectious Diseases).[18,19]

3. References

[1] A. Shafir, M. P. Power, G. D. Whitener, J. Arnold, *Organometallics* **2000**, *19*, 3978–3982.
[2] S. Straub, P. Brünker, J. Lindner, P. Vöhringer, *Phys. Chem. Chem. Phys.* **2018**, *20*, 21390–21403.
[3] T. Unruh, L. I. Domenianni, P. Vöhringer, *Mol. Phys.* **2021**, *119*, e1964043.
[4] S. Straub, L. I. Domenianni, J. Lindner, P. Vöhringer, *J. Phys. Chem. B* **2019**, *123*, 7893–7904.
[5] F. Neese, *WIREs Comput. Mol. Sci.* **2022**, *12*, e1606.
[6] J. Steinmetzer, S. Kupfer, S. Gräfe, *Int. J. Quantum Chem.* **2021**, *121*, e26390.
[7] J. P. Perdew, M. Ernzerhof, K. Burke, *J. Chem. Phys.* **1996**, *105*, 9982–9985.
[8] F. Weigend, R. Ahlrichs, *Phys. Chem. Chem. Phys.* **2005**, *7*, 3297.
[9] F. Weigend, *Phys. Chem. Chem. Phys.* **2006**, *8*, 1057–1065.

[10] E. Caldeweyher, S. Ehlert, A. Hansen, H. Neugebauer, S. Spicher, C. Bannwarth, S. Grimme, *J. Chem. Phys.* **2019**, *150*, 154122.
[11] V. Barone, M. Cossi, *J. Phys. Chem. A* **1998**, *102*, 1995–2001.
[12] D. A. Pantazis, X.-Y. Chen, C. R. Landis, F. Neese, *J. Chem. Theory Comput.* **2008**, *4*, 908–919.
[13] C. Adamo, V. Barone, *J. Chem. Phys.* **1999**, *110*, 6158–6170.
[14] B. Helmich-Paris, B. de Souza, F. Neese, R. Izsák, *J. Chem. Phys.* **2021**, *155*, 104109.
[15] J. Zheng, X. Xu, D. G. Truhlar, *Theor. Chem. Acc.* **2011**, *128*, 295–305.
[16] B. de Souza, G. Farias, F. Neese, R. Izsák, *J. Chem. Theory Comput.* **2019**, *15*, 1896–1904.
[17] T. Yanai, D. P. Tew, N. C. Handy, *Chem. Phys. Lett.* **2004**, *393*, 51–57.
[18] E. F. Pettersen, T. D. Goddard, C. C. Huang, E. C. Meng, G. S. Couch, T. I. Croll, J. H. Morris, T. E. Ferrin, *Protein Sci. Publ. Protein Soc.* **2021**, *30*, 70–82.
[19] A. J. Schaefer, V. M. Ingman, S. E. Wheeler, *J. Comput. Chem.* **2021**, *42*, 1750–1754.

4. Details on Complete Active Space Calculations

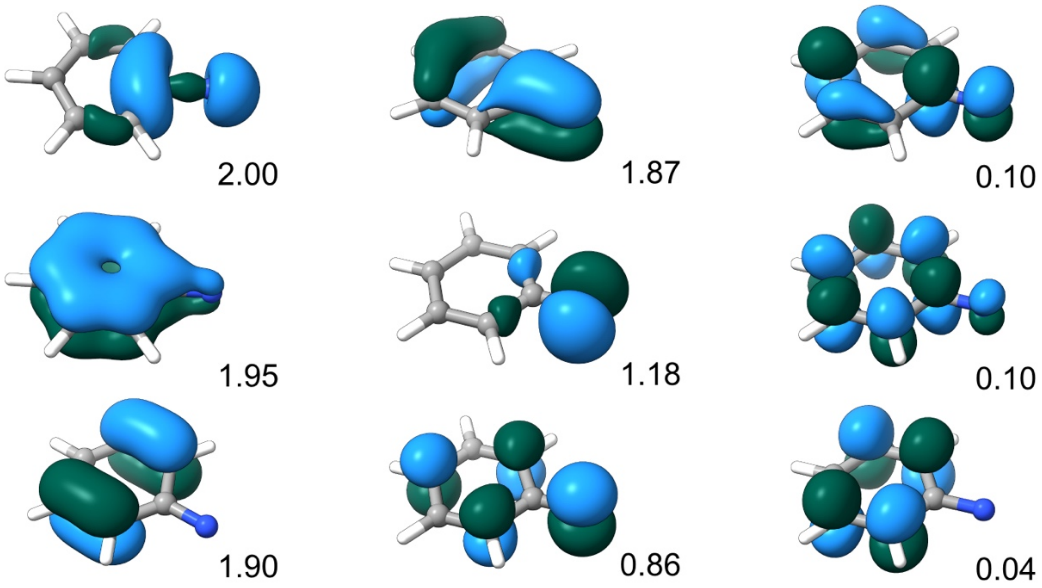

Figure S1. Active space orbitals (contour value 0.05) and their occupation numbers of the Ph-N SA-CASSCF(10,9)-NEVPT2/ma-def2-TZVP, CPCM(MeCN) calculations (cf. Fig 1 in the main text).

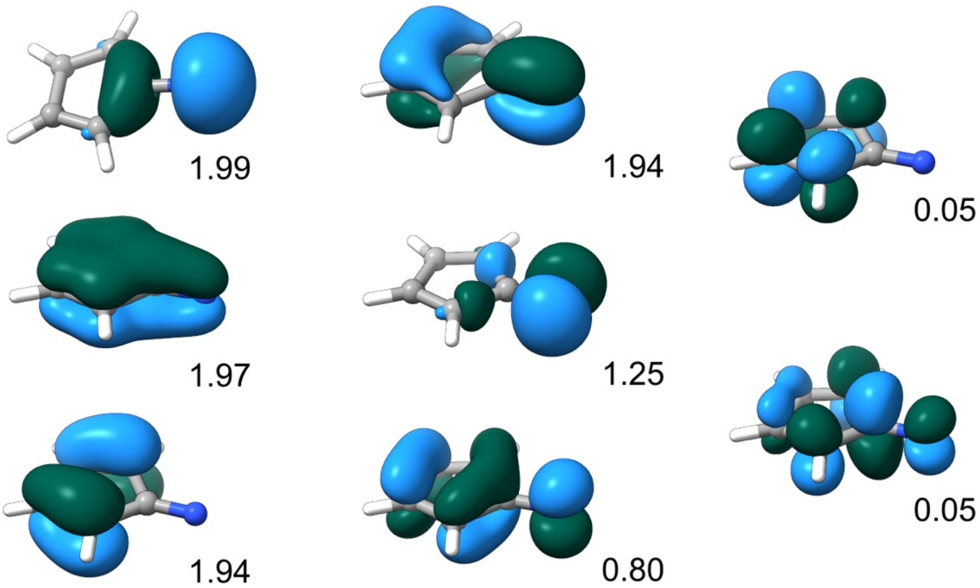

Figure S2. Active space orbitals (contour value 0.05) and their occupation numbers of the $[Cp\text{-}N]^-$ SA-CASSCF(10,8)-NEVPT2/ma-def2-TZVP, CPCM(MeCN) calculations (cf. Fig 1 in the main text).

Table S1. Computed spin state energies for Ph-N as Final Single Point Energies (FSPE, D4-PBE0/ma-def2-TZVP//D4-PBE/def2-TZVP, CPCM(MeCN)) and values relative to the ground state (ΔFSPE) compared to the SA-CASSCF(10,9)/ma-def2-TZVP//D4-PBE/def2-TZVP, CPCM(MeCN) (see Figure S1 for the active space orbitals) computed spin state energies ($FSPE_{CASSCF}$) and with added NEVPT2 correction ($FSPE_{NEVPT2}$) (cf. bold entries and Figure 1 in the main text). For comparison all FSPE values were also calculated with the regular def2-TZVP basis set.

State	Basis set	FSPE [Eh]	ΔFSPE [kJ/mol]	$FSPE_{CASSCF}$ [Eh]	$ΔFSPE_{CASSCF}$ [kJ/mol]	$FSPE_{NEVPT2}$ [Eh]	$ΔFSPE_{NEVPT2}$ [kJ/mol]
$^3A_2$	def2-TZVP	-286.0839	0.0	-284.6951	0.0	-285.6774	0.0
	**ma-def2-TZVP**	-286.0845	**0.0**	-284.6953	**0.0**	-285.6786	**0.0**
$^1A_2$	def2-TZVP	-286.0710	33.9	-284.6647	79.6	-285.6484	76.1
	**ma-def2-TZVP**	-286.0717	**33.7**	-284.6651	**79.3**	-285.6498	**75.8**
$^1A_1$	def2-TZVP	-286.0364	124.6	-284.6285	174.7	-285.6320	119.1
	**ma-def2-TZVP**	-286.0381	**121.7**	-284.6290	**174.0**	-285.6337	**117.9**

Table S2. Computed spin state energies for [Cp-N]- as Final Single Point Energies (FSPE, D4-PBE0/ma-def2-TZVP//D4-PBE/def2-TZVP, CPCM(MeCN)) and values relative to the ground state (ΔFSPE) compared to the SA-CASSCF(10,8)/ma-def2-TZVP//D4-PBE/def2-TZVP), CPCM(MeCN) (see Figure S2 for the active space orbitals computed spin state energies ($FSPE_{CASSCF}$) and with added NEVPT2 correction ($FSPE_{NEVPT2}$) (cf. bold entries and Figure 1 in the main text). For comparison all FSPE values were also calculated with the regular def2-TZVP basis set.

State	Basis set	FSPE [Eh]	ΔFSPE [kJ/mol]	$FSPE_{CASSCF}$ [Eh]	$ΔFSPE_{CASSCF}$ [kJ/mol]	$FSPE_{NEVPT2}$ [Eh]	$ΔFSPE_{NEVPT2}$ [kJ/mol]
$^3A_2$	def2-TZVP	-247.5070	20.0	-246.2435	67.7	-247.1582	45.2
	**ma-def2-TZVP**	-247.5092	26.2	-246.2445	72.7	-247.1615	49.5
$^1A_2$	def2-TZVP	-247.5068	20.7	-246.2232	121.0	-247.1391	95.5
	**ma-def2-TZVP**	-247.4999	50.9	-246.2247	124.7	-247.1427	98.8
$^1A_1$	def2-TZVP	-247.5146	0.0	-246.2693	0.0	-247.1754	0.0
	**ma-def2-TZVP**	-247.5192	0.0	-246.2722	0.0	-247.1803	0.0

Table S3. Computed spin state energies for 1-azido-1'-nitrenoferrocene as Final Single Point Energies (FSPE, D4-PBE0/ma-def2-TZVP//D4-PBE/def2-TZVP, CPCM(MeCN)) and values relative to the ground state (ΔFSPE) (cf. bold entries and Figure 1 in the main text). For comparison all FSPE values were also calculated with the regular def2-TZVP basis set.

State	Basis	**FSPE [Eh]**	**ΔFSPE [kJ/mol]**
$^3A_2$	def2-TZVP	-1867.8144	0.0
	**ma-def2-TZVP**	-1867.8159	**0.0**
$^1A_2$	def2-TZVP	-1867.8088	14.9
	**ma-def2-TZVP**	-1867.8104	**14.4**
$^1A_1$	def2-TZVP	-1867.7913	60.8
	**ma-def2-TZVP**	-1867.7936	**58.5**

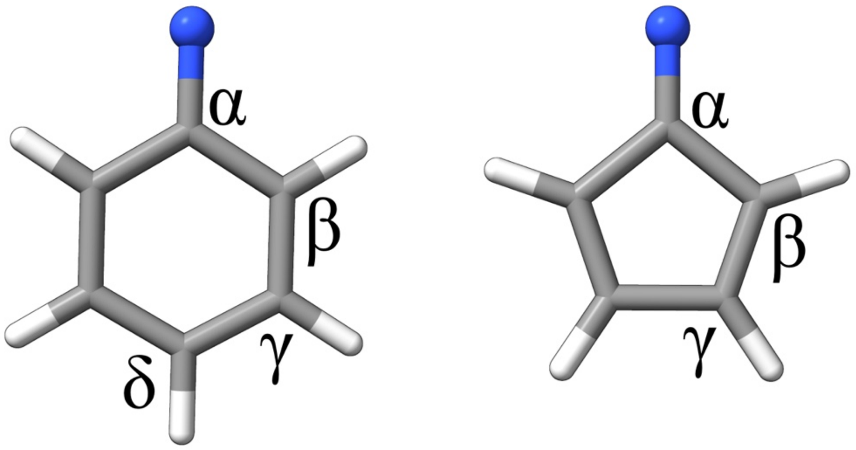

Figure S3. Atom labels for the assignment of the Loewdin spin population numbers in Table S4 and Table S5 for the Ph-N (left) and $[Cp\text{-}N]^-$/1-azido-1'-nitrenoferrocene (right).

Table S4. DFT computed Loewdin spin populations (D4-PBE0/ma-def2-TZVP//D4-PBE/def2-TZVP, CPCM(MeCN)) for the open-shell singlet states ($^1A_2$) of the single point calculations in Tables S1-S3 (cf. Figure 1 in the main text). The molecules are oriented such that p_z is perpendicular to the plane of the ring, p_y is oriented along the N-C bond, and p_x lies in the plane of the molecule.

$^1A_2$	Ph-N	$[Cp\text{-}N]^-$	1-azido-1'-nitrenoferrocene
N	0.36<br>(-0.45 p_z, 0.79 p_x)	0.44<br>(-0.33 p_z, 0.78 p_x)	0.72<br>(0.11 p_z, 0.64 p_x)
C_α	-0.23	-0.02	0.09
C_β,C_β'	-0.15	-0.08	0.08
C_γ, C_γ'	0.05	-0.13	0.04
C_δ	0.08	-	-
Fe	-	-	-1.09

Table S5. DFT computed Loewdin spin populations (D4-PBE0/ma-def2-TZVP//D4-PBE/def2-TZVP, CPCM(MeCN)) for the triplet states ($^3A_1$) of the single point calculations in Tables S1-S3 (cf. Figure 1 in the main text).

$^3A_2$	Ph-N	$[Cp\text{-}N]^-$	1-azido-1'-nitrenoferrocene
N	1.36	1.22	1.14
C_α	0.09	0.19	0.09
C_β,C_β'	0.21	0.17	0.08
C_γ, C_γ'	-0.04	0.12	0.01
C_δ	0.21	-	-
Fe	-	-	0.60

5. Difference densities and natural transition orbitals

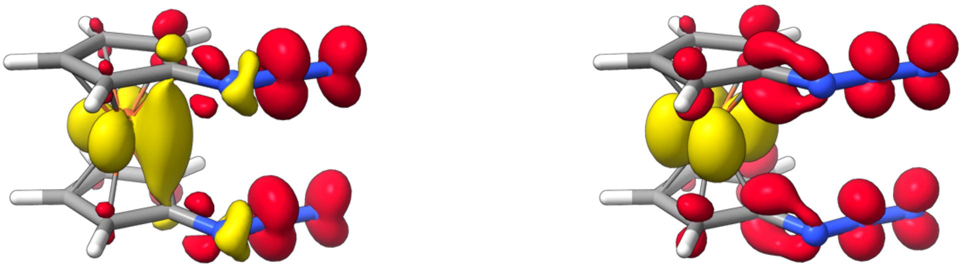

Figure S4. Singlet (S_9 96%, left) and triplet (T_{16} 3%, right) difference densities (yellow: density loss, red: density gain, contour value 0.0025, SOCME(T_{16}, S_9) 201.7 cm^{-1}) for the MLCT transition **A** (cf. Figure 3 in the main text).

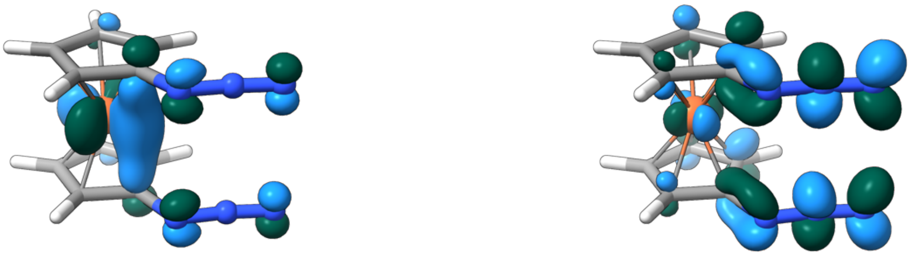

Figure S5. Dominant natural transition donor (left) and acceptor (right) orbitals (84%) of the major singlet root contributing to the MLCT transition **A** (cf. Figure 3 in the main text).

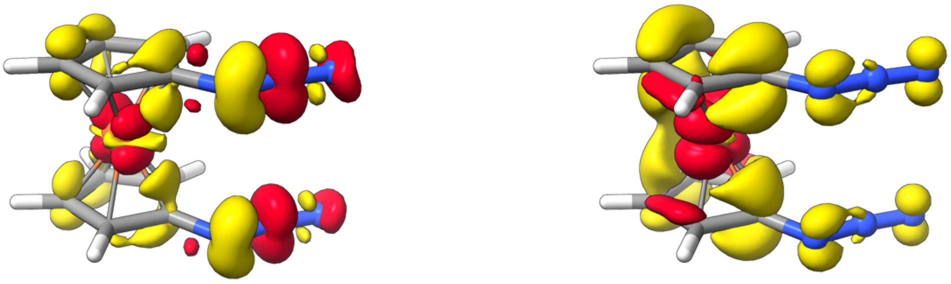

Figure S6. Singlet (S_{10} 96%, left) and triplet (T_{18} 4%, right) difference densities (yellow: density loss, red: density gain, contour value 0.0025, SOCME(T_{18}, S_{10}) 88.1 cm^{-1}) for the LMCT/LLCT transition **B** (cf. Figure 3 in the main text).

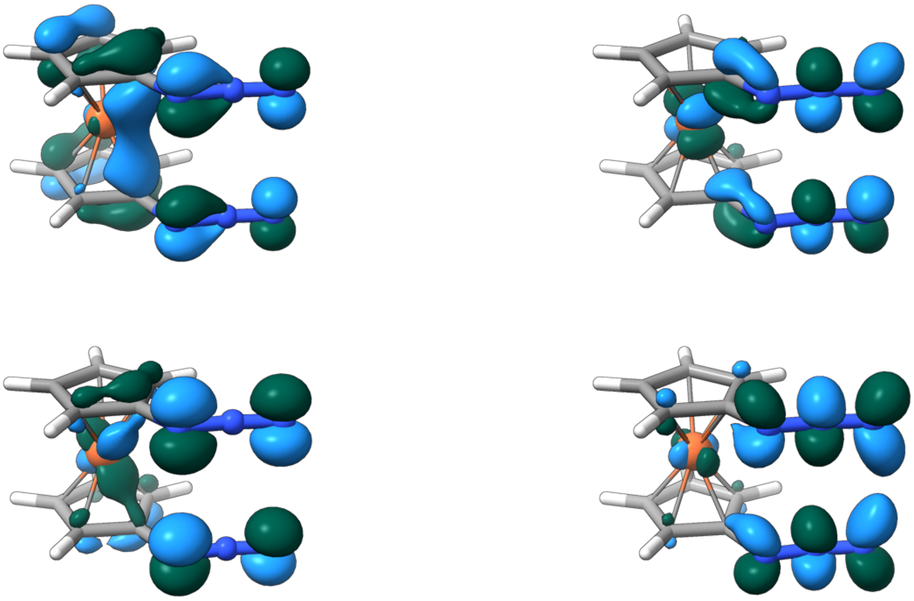

Figure S7. Dominant natural transition donor (left) and acceptor (right) orbitals (65% top, 15% bottom) of the major singlet roots contributing to the LMCT/LLCT transition **B** (cf. Figure 3 in the main text).

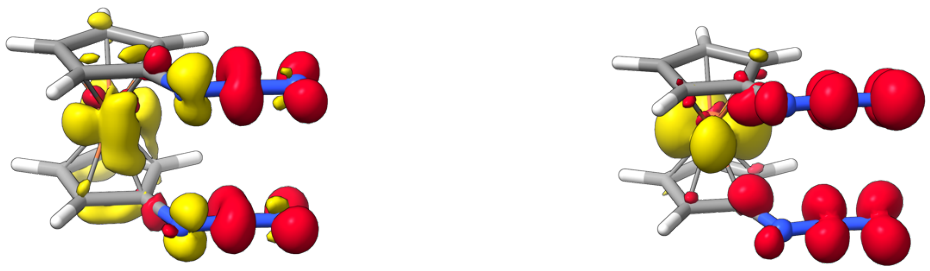

Figure S8. Singlet (S_{17} 74%, left) and triplet (T_{24} 8%, right) difference densities (yellow: density loss, red: density gain, contour value 0.0025, SOCME(T_{24}, S_{17}, S_{18}) 23.7 cm^{-1}) for the MLCT/LLCT transition **C** (cf. Figure 3 in the main text).

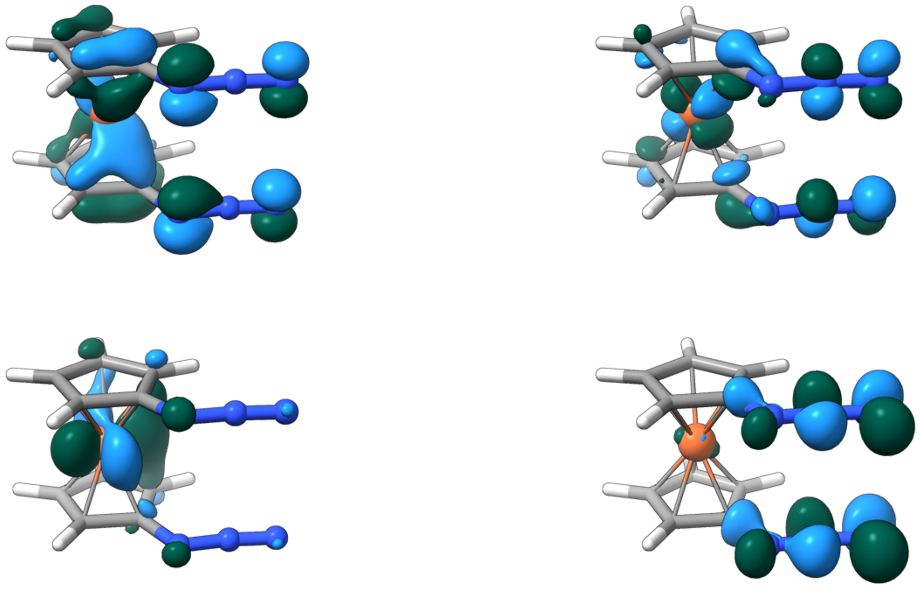

Figure S9. Dominant natural transition donor (left) and acceptor (right) orbitals (46% top, 31% bottom) of the major singlet roots contributing to the MLCT/LLCT transition **C** (cf. Figure 3 in the main text).

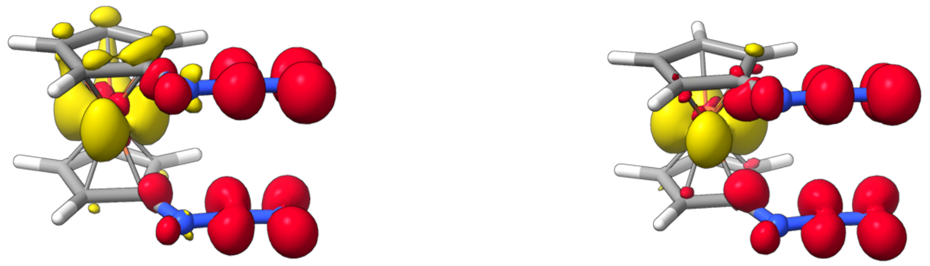

Figure S10. Singlet (S_{18} 61%, left) and triplet (T_{24} 14%, right) difference densities (yellow: density loss, red: density gain, contour value 0.0025, SOCME(T_{24}, S_{17}, S_{18}) 49.3 cm^{-1}) for the MLCT transition **D** (cf. Figure 3 in the main text).

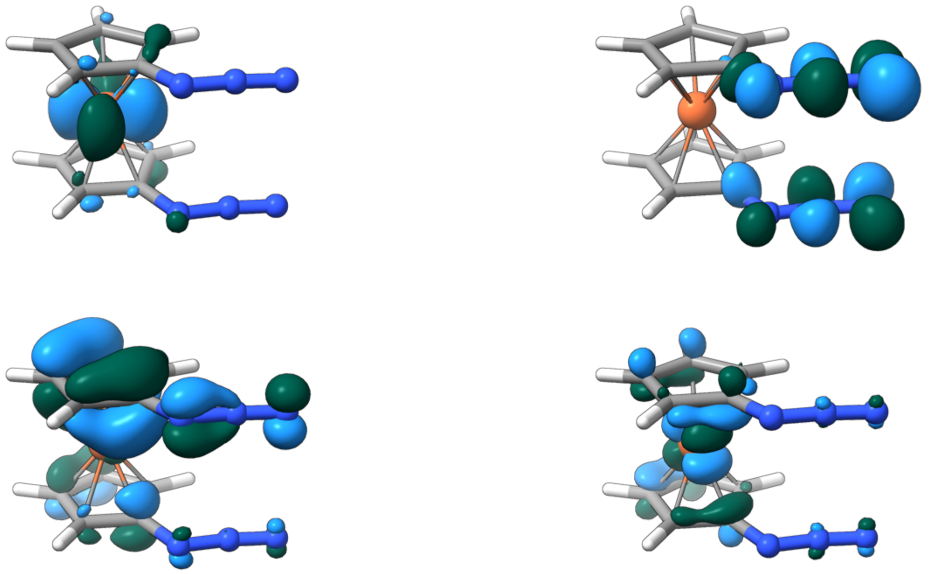

Figure S11. Dominant natural transition donor (left) and acceptor (right) orbitals (67% top, 20% bottom) of the major singlet roots contributing to the MLCT transition **D** (cf. Figure 3 in the main text).

The low-intensity band centered at 447 nm in the experimental spectrum (cf. Figure 3 in the main text) consists of spin-pure metal-centered d-d transitions.

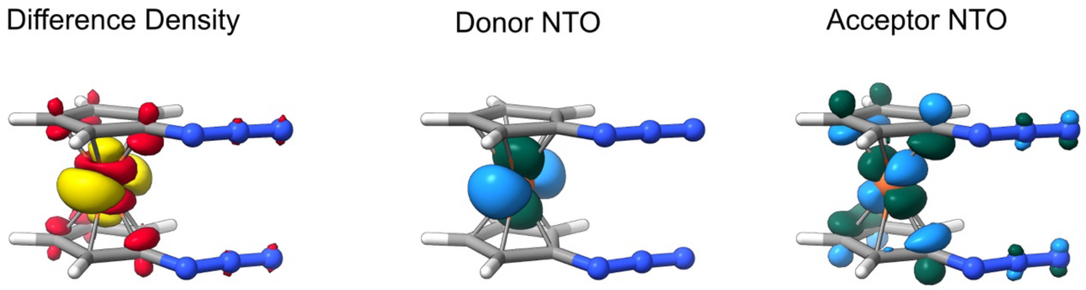

Figure S12. Difference density and natural transition orbitals (NTOs) of one of the d-d transitions contributing to the feature at 447 nm in the experimental UV-Vis spectrum (cf. Figure 3 in the main text).

6. Exploration of the potential energy landscape in the ground state and excited states

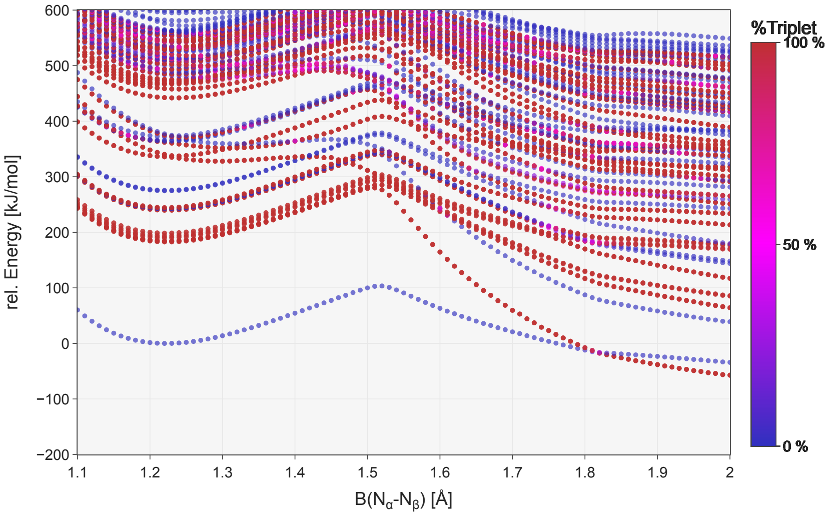

Figure S13. 1,1'-Diazidoferrocene relaxed potential energy surface scan (D4-CAM-B3LYP/def2-TZVP//D4-PBE/def2-SVP incl. SOMF) along one N-N_2 bond length.

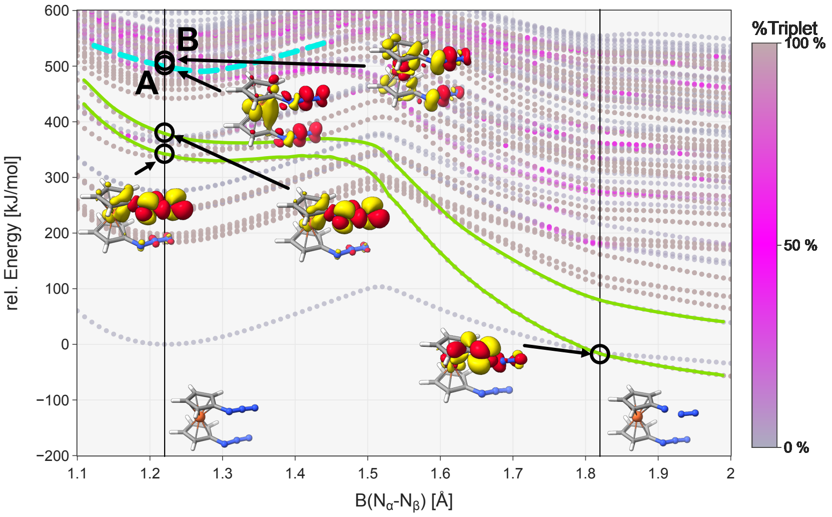

Figure S14. 1,1'-Diazidoferrocene relaxed potential energy surface scan (D4-CAM-B3LYP/def2-TZVP//D4-PBE/def2-SVP incl. SOMF) along one N-N_2 bond length. Adiabatic states (cf. discussion in the main text) are marked green and cyan, the inserts show geometries and difference densities (yellow: density loss, red: density gain, contour value 0.0025) of important scan steps. All difference densities make up at least 95% of the state character.

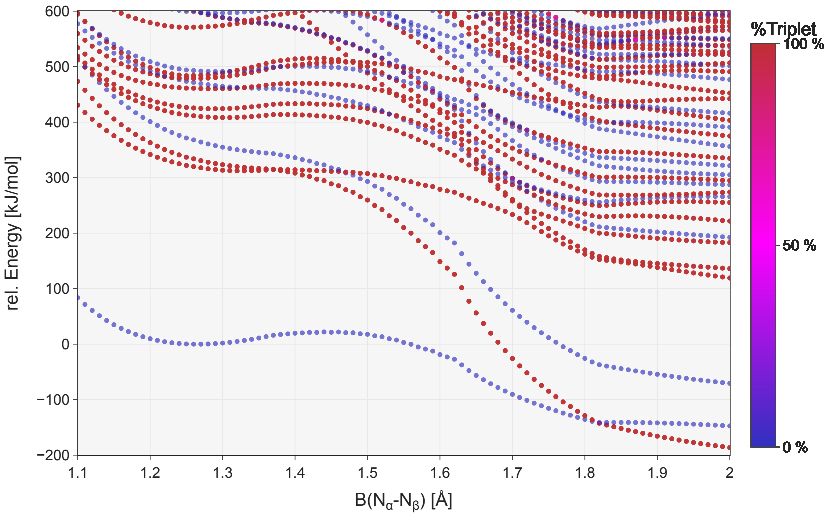

Figure S15. Phenylazide relaxed potential energy surface scan (D4-CAM-B3LYP/def2-TZVP//D4-PBE/def2-SVP incl. SOMF) along the N-N_2 bond length.

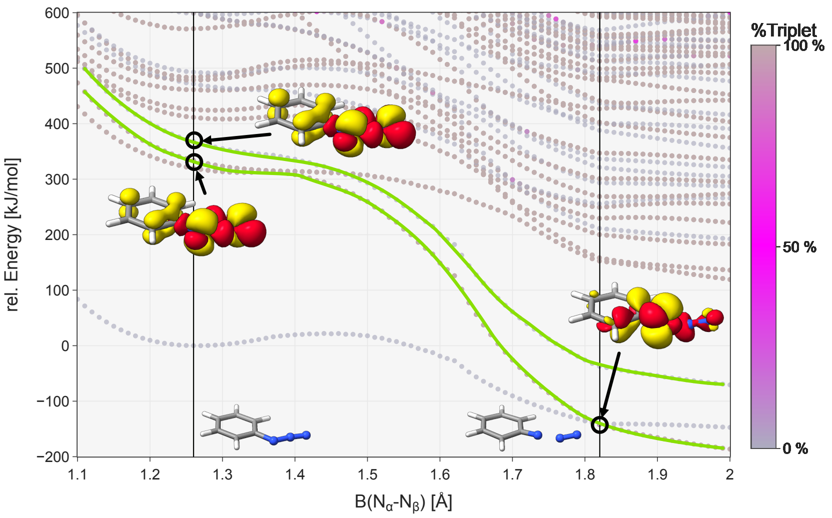

Figure S16. Phenylazide relaxed potential energy surface scan (D4-CAM-B3LYP/def2-TZVP//D4-PBE/def2-SVP incl. SOMF) along the N-N_2 bond length. Adiabatic states (cf. discussion in the main text) are marked green, the inserts show geometries and difference densities (yellow: density loss, red: density gain, contour value 0.0025) of important scan steps. All difference densities make up at least 99% of the state character.

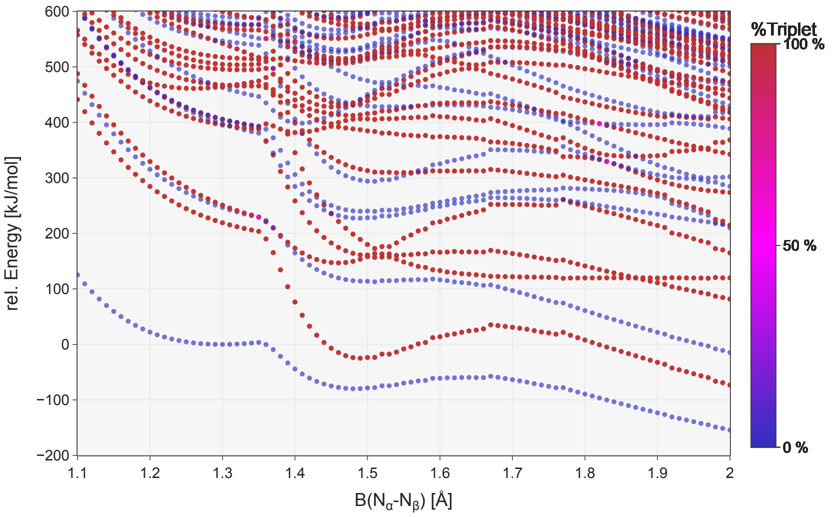

Figure S17. Cyclopentadienylazide anion relaxed potential energy surface scan (D4-CAM-B3LYP/def2-TZVP//D4-PBE/def2-SVP incl. SOMF) along the N-N_2 bond length. Notice that the second ground state minimum at 1.48 Å is not a true minimum but rather an artefact of the constrained scan coordinate that would relax along orthogonal dimensions in an unconstrained geometry optimization.

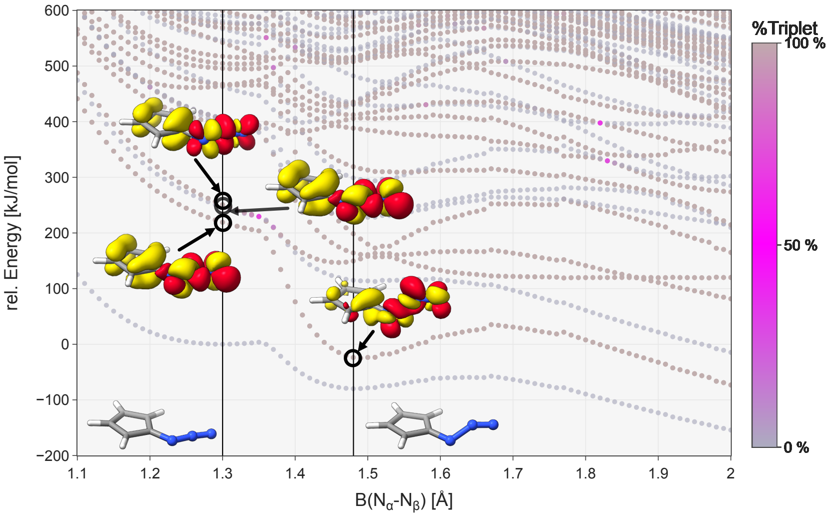

Figure S18. Cyclopentadienylazide anion relaxed potential energy surface scan (D4-CAM-B3LYP/def2-TZVP//D4-PBE/def2-SVP incl. SOMF) along the N-N_2 bond length. Inserts show geometries and difference densities (yellow: density loss, red: density gain, contour value 0.0025) of important scan steps. All difference densities make up at least 99% of the state character. Notice that the second ground state minimum at 1.48 Å is not a true minimum but rather an artefact of the constrained scan coordinate that would relax along orthogonal dimensions.

Conformational Analysis

Geometries were optimized with D4-PBE/def2-TZVP, CPCM(MeCN) and confirmed to be a local minimum via a frequency calculation.

In the ferrocenyl diazide complex, the two Cp-N_3 ligands were expected to a have a low barrier for rotation long the Cp-Fe-Cp axis. To take this flexibility into account, 10 conformational isomers (each rotated along the Cp-Fe-Cp axis by +36°) were computed. For the ground state, 10 additional conformers were included where the two azide moieties are oriented *anti* relative to each other.

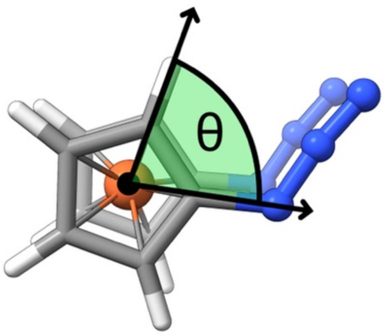

Figure S19. Rotation axis for the conformer generation. The axis of rotation is aligned through the center of both Cp-N_3 rings and the iron center. The rotational conformers are generated by rotating one Cp-N_3 ligand about this axis in 36° steps, in the example shown θ = 72°.

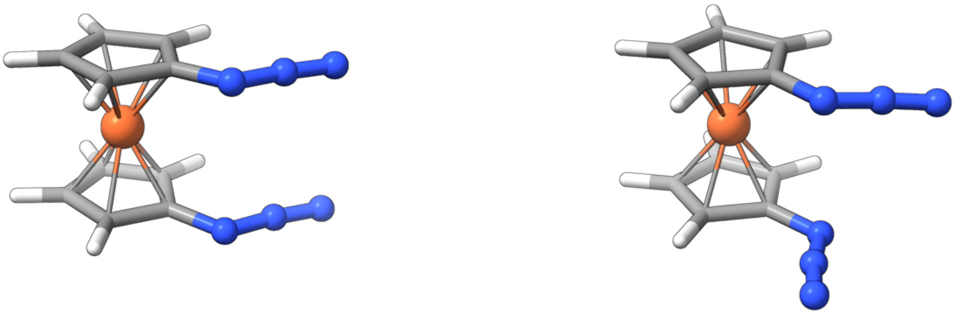

Figure S20. Ferrocenyl diazide ground state structure with the azide moieties *syn* to each other (left) and the corresponding *anti* structure (right).

Some conformers (mostly with a staggered orientation of the two Cp-N_3 rings) exhibited a single imaginary frequency in the vibrational analysis. Thus, they correspond to the transition states between stable conformers and were not included in the Boltzmann distribution. Certain conformations could not be optimized as the geometry optimizations would simply converge to a neighboring local minimum and were also not included.

Since the experimental measurements were taken at room temperature, a temperature of 298 K was assumed to compute the Boltzmann distribution of the conformers.

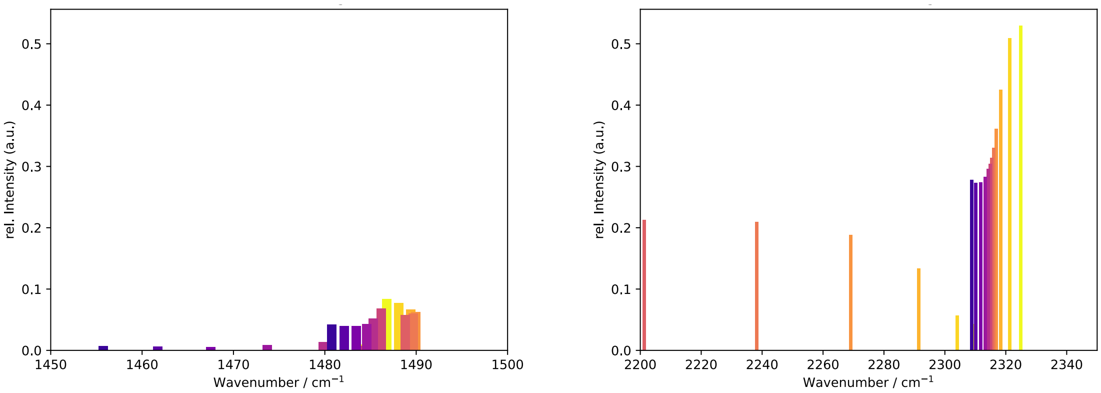

Figure S21. Relaxed surface scan (D4-PBE/def2-TZVP, CPCM(MeCN)) of one N_α-N_β-N_γ bending angle of the 1,1'-diazidoferrocene ground state from 175° (yellow) to 120° (blue). Shown are the spectral IR regions of the CN/CH-stretching region (left) and the azide stretching region (right). With increasing N_α-N_β-N_γ angle, the vibrational modes successively decouple. In the azide stretching region, the spectral feature of the bending azide can be observed to rapidly move to lower energies as the structural motif changes from a linear azide to a more isolated nitrogen-nitrogen double bond. Importantly, the decoupling of the azide stretching modes leads to a redshift of the undisturbed azide stretching mode of about 20 cm^{-1}.

Table S6. Conformational analysis data of the ferrocenyl diazide ground state (S = 0), computed at 298 K. The 'relative azide orientation' refers to Figure S8.

**Rotation [°] along Cp-Fe-Cp axis**	**relative azide orientation**	**stable?**	**Calc. Gibbs free energy (GFE) [E_h]**	**rel. GFE [kJ/mol]**	**Boltzmann probability partition (N_i)**	**Boltzmann factor**
**0**	syn	Yes	-1977.1910	0.0	1.0	27.9 %
**36**	syn	-	-	-	-	0.0 %
**72**	syn	Yes	-1977.1898	3.3	0.3	7.5 %
**108**	syn	imag. freq.	-1977.1874	9.5	-	0.0 %
**144**	syn	Yes	-1977.1895	4.0	0.2	5.6 %
**180**	syn	imag. freq.	-1977.1872	10.0	-	0.0 %
**216**	syn	Yes	-1977.1895	4.0	0.2	5.6 %
**252**	syn	imag. freq.	-1977.1875	9.3	-	0.0 %
**288**	syn	Yes	-1977.1898	3.2	0.3	7.6 %
**324**	syn	-	-	-	-	0.0 %
**0**	anti	Yes	-1977.1907	0.9	0.7	19.3 %
**36**	anti	-	-	-	-	0.0 %
**72**	anti	Yes	-1977.1896	3.7	0.2	6.2 %
**108**	anti	imag. freq.	-1977.1874	9.4	-	0.0 %
**144**	anti	Yes	-1977.1894	4.2	0.2	5.1 %
**180**	anti	-	-	-	-	0.0 %
**216**	anti	Yes	-1977.1895	4.0	0.2	5.5 %
**252**	anti	imag. freq.	-1977.1875	9.2	-	0.0 %
**288**	anti	Yes	-1977.1900	2.6	0.3	9.8 %
**324**	anti	imag. freq.	-1977.1882	7.5	-	0.0 %

Table S7. Computed azide- and CN/CH-stretching IR absorption bands of the conformational analysis of the ferrocenyl diazide ground state (S = 0).

**Rotation [°] along Cp-Fe-Cp axis**	**Azide-stretch band 1**		**Azide-stretch band 2**		**CN/CH-stretch band 1**		**CN/CH-stretch band 2**	
	Energy [cm^{-1}]	Extinction coefficient [$M^{-1}cm^{-1}$]	Energy [cm^{-1}]	Extinction coefficient [$M^{-1}cm^{-1}$]	Energy [cm^{-1}]	Extinction coefficient [$M^{-1}cm^{-1}$]	Energy [cm^{-1}]	Extinction coefficient [$M^{-1}cm^{-1}$]
**0**	2168.13	0.520	2150.61	0.039	1446.88	0.069	1445.73	0.006
**36**	2168.27	0.520	2150.85	0.039	1446.81	0.070	1445.70	0.006
**72**	2164.32	0.363	2157.18	0.234	1445.31	0.049	1444.03	0.040
**108**	2164.03	0.192	2155.93	0.443	1447.09	0.028	1444.39	0.062
**144**	2164.20	0.054	2155.49	0.606	1446.64	0.008	1443.84	0.090
**180**	2164.55	0.000	2156.30	0.661	1449.84	0.000	1443.62	0.101
**216**	2164.19	0.054	2155.47	0.607	1446.63	0.008	1443.84	0.089
**252**	2164.01	0.191	2155.93	0.440	1447.08	0.028	1444.36	0.062
**288**	2164.52	0.381	2157.36	0.211	1444.89	0.048	1443.78	0.040
**324**	2168.39	0.518	2150.40	0.038	1446.23	0.070	1445.18	0.006
**0**	2166.36	0.316	2155.24	0.277	1446.08	0.070	1443.93	0.011
**36**	2166.14	0.321	2155.13	0.272	1445.95	0.070	1443.79	0.011
**72**	2164.08	0.029	2156.85	0.620	1445.46	0.028	1444.00	0.067
**108**	2164.12	0.004	2156.13	0.657	1447.02	0.008	1444.45	0.085
**144**	2164.21	0.084	2155.53	0.579	1446.14	0.001	1443.87	0.097
**180**	2164.27	0.085	2155.59	0.578	1446.17	0.000	1443.95	0.098
**216**	2164.39	0.410	2155.34	0.204	1447.26	0.025	1443.95	0.066
**252**	2164.25	0.522	2155.53	0.054	1447.00	0.050	1444.35	0.035
**288**	2166.41	0.533	2156.19	0.039	1444.89	0.068	1444.18	0.016
**324**	2165.36	0.460	2153.37	0.127	1446.37	0.006	1445.42	0.071

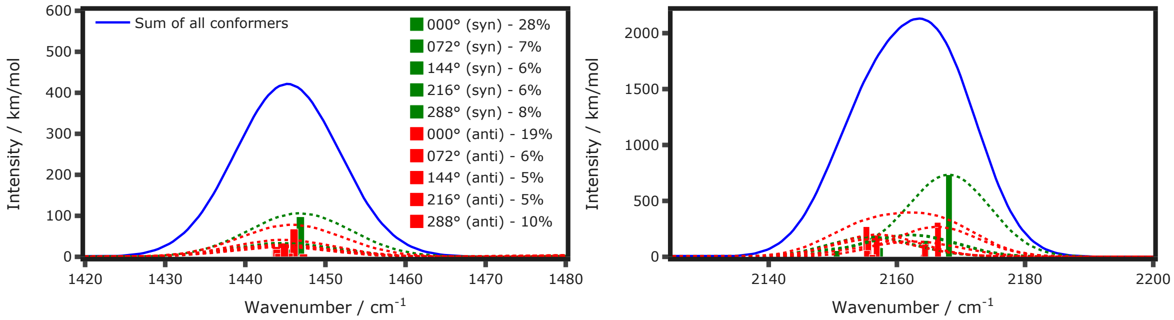

Figure S22. Boltzmann-weighted CN/CH-stretching (left) and azide-stretching (right) bands of the ferrocenyl diazide ground state (S = 0) (cf. Figure 5 in the main text).

Table S8. Conformational analysis data of the proposed intermediate (S = 1), computed at 298 K.

Rotation [°] along Cp-Fe-Cp axis	stable?	Calc. Gibbs free energy (GFE) [E_h]	rel. GFE [kJ/mol]	Boltzmann probability partition (N_i)	Boltzmann factor
**0**	Yes	-1977.1457	0.0	1.0	45.1 %
**36**	-	-	-	-	0.0 %
**72**	Yes	-1977.1436	5.6	0.1	4.8 %
**108**	-	-	-	-	0.0 %
**144**	Yes	-1977.1434	6.0	0.1	4.0 %
**180**	imag. Freq.	-1977.1412	11.9	-	0.0 %
**216**	-	-	-	-	0.0 %
**252**	imag. Freq.	-1977.1421	9.4	-	0.0 %
**288**	Yes	-1977.1436	5.5	0.1	4.9 %
**324**	Yes	-1977.1456	0.2	0.9	41.3 %

Table S9. Computed azide- and CN/CH-stretching IR absorption bands of the conformational analysis of the proposed intermediate (S = 1).

Rotation [°] along Cp-Fe-Cp axis	Azide-stretch band 1		Azide-stretch band 2		CN/CH-stretch band 1		CN/CH-stretch band 2	
	Energy [cm^{-1}]	Extinction coefficient [$M^{-1}cm^{-1}$]	Energy [cm^{-1}]	Extinction coefficient [$M^{-1}cm^{-1}$]	Energy [cm^{-1}]	Extinction coefficient [$M^{-1}cm^{-1}$]	Energy [cm^{-1}]	Extinction coefficient [$M^{-1}cm^{-1}$]
**0**	2169.37	0.258	1709.50	0.0358	1455.57	0.1039	1439.09	0.0557
**36**	2170.11	0.256	1708.65	0.0357	1455.75	0.1028	1438.66	0.0560
**72**	2170.04	0.347	1713.07	0.0390	1461.66	0.0914	1439.26	0.0968
**108**	2170.20	0.343	1713.09	0.0390	1461.69	0.0907	1438.88	0.0947
**144**	2169.02	0.353	1710.53	0.0413	1451.34	0.0636	1435.75	0.1118
**180**	2170.04	0.364	1711.97	0.0461	1464.14	0.0378	1437.24	0.1602
**216**	-	-	-	-	-	-	-	-
**252**	2170.19	0.303	1714.93	0.0428	1458.29	0.0843	1440.18	0.1080
**288**	2171.13	0.283	1712.98	0.0421	1459.99	0.0911	1438.90	0.0925
**324**	2169.43	0.258	1708.68	0.0361	1456.06	0.1029	1438.94	0.0561

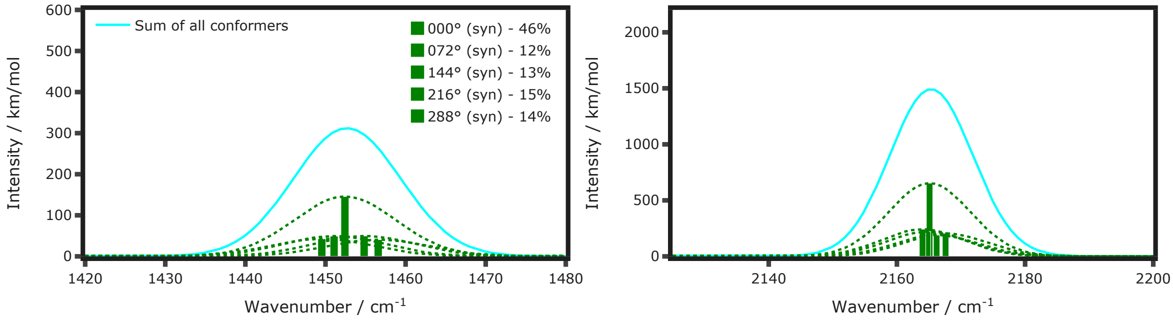

Figure S23. Boltzmann-weighted CN/CH-stretching (left) and azide-stretching (right) bands of the proposed intermediate (S = 1) (cf. Figure 5 in the main text).

Table S10. Conformational analysis data of the ferrocenylazide nitrene (*S* = 1), computed at 298 K.

Rotation [°] along Cp-Fe-Cp axis	stable?	Calc. Gibbs free energy (GFE) [E_h]	rel. GFE [kJ/mol]	Boltzmann probability partition (N_i)	Boltzmann factor
**0**	Yes	-1867.7289	0.0	1.0	46.1 %
**36**	-	-	-	-	0.0 %
**72**	Yes	-1867.7276	3.4	0.3	11.7 %
**108**	-	-	-	-	0.0 %
**144**	Yes	-1867.7277	3.1	0.3	12.9 %
**180**	-	-	-	-	0.0 %
**216**	Yes	-1867.7279	2.8	0.3	14.9 %
**252**	-	-	-	-	0.0 %
**288**	Yes	-1867.7278	2.9	0.3	14.3 %
**324**	-	-	-	-	0.0 %

Table S11. Computed azide- and CN/CH-stretching IR absorption bands of the conformational analysis of the ferrocenylazide nitrene (*S* = 1).

Rotation [°] along Cp-Fe-Cp axis	Azide-stretch band 1		CN/CH-stretch band 1		CN/CH-stretch band 2	
	Energy [cm^{-1}]	Extinction coefficient [$M^{-1}cm^{-1}$]	Energy [cm^{-1}]	Energy [cm^{-1}]	Extinction coefficient [$M^{-1}cm^{-1}$]	Energy [cm^{-1}]
**0**	2165.10	0.2807	1452.43	0.0626	1405.43	0.0001
**36**	2164.82	0.2804	1451.60	0.0634	1405.94	0.0002
**72**	2166.20	0.3214	1456.58	0.0687	1402.99	0.0002
**108**	2166.21	0.3230	1456.39	0.0695	1404.25	0.0002
**144**	2164.77	0.3381	1449.54	0.0653	1399.29	0.0001
**180**	2164.79	0.3394	1448.72	0.0669	1399.56	0.0002
**216**	2164.00	0.3225	1451.07	0.0657	1400.46	0.0001
**252**	2164.20	0.3229	1451.13	0.0658	1401.07	0.0001
**288**	2167.59	0.2834	1454.82	0.0685	1405.83	0.0002
**324**	2164.88	0.2848	1452.38	0.0629	1405.86	0.0001

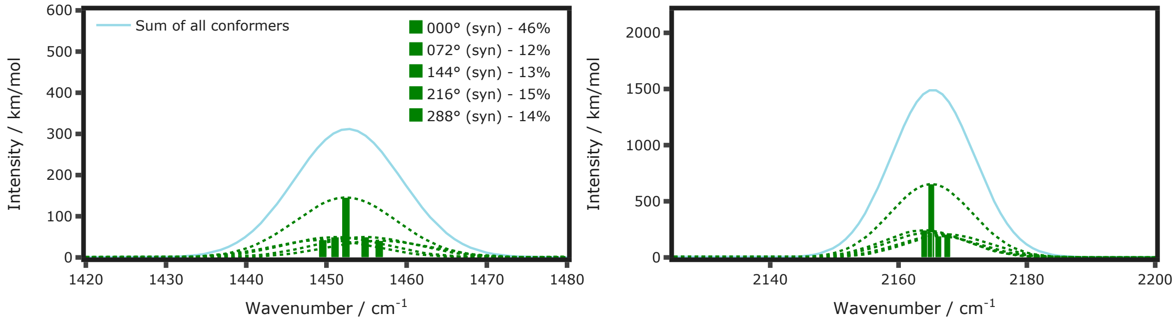

Figure S24. Boltzmann-weighted CN/CH-stretching (left) and azide-stretching (right) bands of the ferrocenylazide nitrene (*S* = 1) (cf. Figure 5 in the main text).

Table S12. Calculated Gibbs free energies for the proposed reaction energy profile. The open-shell singlet, OSS, was obtained with BS-DFT.

		Electronic energy E(el)$_{PBE0-D4}$ [E$_h$]	Gibbs free energy ΔG$_{PBE-D4}$ [E$_h$]	GFE-Correction to the electronic energy [E$_h$]	E(el)$_{PBE0-D4}$ + GFE-Correction [E$_h$]	relative corrected ΔG [kJ/mol]
**Ground State**	*S*=0	-1977.2671	-1977.1911	0.1296	-1977.1375	**0.0**
	S=1	-1977.2358	-1977.1403	0.1235	-1977.1123	66.2
**Intermediate**	*S*=0	-1977.1948	-1977.1363	0.1262	-1977.0686	180.7
	S=0 (OSS)	-1977.2256	-1977.1450	0.1260	-1977.0996	99.4
	S=1	-1977.2262	-1977.1457	0.1257	-1977.1006	**96.8**
**Transition State**	*S*=0	—	—	—	-1977.0529	222.1
	S=1	—	—	—	-1977.0948	**111.9**
**Product**	*S*=0	-1867.7913	-1867.7156	0.1217	-1867.6696	21.3
	S=1	-1867.8144	-1867.7290	0.1188	-1867.6956	**-47.1**
	N_2 (*S*=0)	-109.4467	-109.4661	-0.0131	-109.4598	—

Table S13. Loewdin spin populations of selected atoms (D4-PBE0/def2-TZVP//D4-PBE/def2-TZVP, CPCM(MeCN)).

		Fe	C	N(α)	N(β)	N(γ)	SUM
**Ground State**	*S*=1	1.89	-0.04	-0.01	0.02	0.05	1.91
**Intermediate**	*S*=0 (OSS)	1.14	-0.05	-0.09	-0.25	-0.59	0.16
	S=1	1.07	-0.02	0.20	0.24	0.64	2.13
**Transition State**	*S*=1	1.19	-0.02	0.42	0.15	0.42	2.16
**Product**	*S*=0 (OSS)	-1.08	0.08	0.72	-	-	-0.28
	S=1	0.60	0.08	1.14	-	-	1.82

Table S14. Calculated Gibbs free energies (D4-PBE0/def2-TZVP, CPCM(MeCN)) and other energies for the proposed reaction energy profile (cf. Figure 4 in the main text). The open-shell singlet, OSS, was obtained with BS-DFT.

		Electronic Energy [E$_h$]	Zero Point Energy [E$_h$]	Total Enthalpy [E$_h$]	Total Entropy Correction (298 K) [E$_h$]	Gibbs free energy G [E$_h$]	relative corrected ΔG [kJ/mol]
**Ground State**	*S*=0	-1977.271381	0.176821	-1977.079750	-0.054832	-1977.1346	**0.0**
	S=1	-1977.240724	0.173013	-1977.051440	-0.059181	-1977.1106	62.9
**Intermediate**	*S*=0					—	—
	S=0 (OSS)	-1977.231538	0.174315	-1977.041952	-0.055582	-1977.0975	97.3
	S=1	-1977.231606	0.174355	-1977.041998	-0.056584	-1977.0986	**94.5**
**Transition State**	*S*=0	-1977.183795	0.171018	-1976.997512	-0.055363	-1977.0529	214.5
	S=1	-1977.225100	0.172016	-1977.037626	-0.057220	-1977.0948	**104.3**
**Product**	*S*=0	-1867.794713	0.166064	-1867.615760	-0.050368	-1867.6661	22.2
	S=1	-1867.817554	0.164374	-1867.639817	-0.052560	-1867.6924	**-46.7**
	N_2 (*S*=0)	-109.447232	0.005664	-109.438262	-0.021727	-109.4600	—

Table S15. Loewdin spin populations of selected atoms (D4-PBE0/def2-TZVP, CPCM(MeCN)).

		Fe	C	N(α)	N(β)	N(γ)	SUM
**Ground State**	*S*=1	1.91	0.06	-0.01	0.01	0.04	2.01
**Intermediate**	*S*=0 (OSS)	1.25	-0.02	-0.12	-0.25	-0.61	0.25
	S=1	1.18	0.01	0.13	0.25	0.62	2.19
**Transition State**	*S*=1	1.19	-0.02	0.42	0.15	0.42	2.16
**Product**	*S*=1	0.65	0.10	1.11	-	-	1.86

Table S16. Selected structural parameters of the singlet ground-state structure and the proposed triplet intermediate structure (D4-PBE0/def2-TZVP, CPCM(MeCN)).

	Ground state (S = 0)	Intermediate (S = 1)
B(C-N1)	1.41	1.33
B(N1-N2)	1.22	1.38
B(N2-N3)	1.12	1.18
A(C-N1-N2)	117.5	110.9
A(N1-N2-N3)	173.9	125.9

Table S17. Vibrational frequencies [cm^{-1}] of the proposed triplet transition state structure (D4-PBE0/def2-TZVP, CPCM(MeCN)).

Mode	Frequency [cm^{-1}]
**0**	0.00
**1**	0.00
**2**	0.00
**3**	0.00
**4**	0.00
**5**	0.00
**6**	-573.57
**7**	20.76
**8**	51.55
**9**	57.08
**10**	107.95
**11**	131.69
**12**	137.04
**13**	153.28
**14**	163.97
**15**	195.13
**16**	213.64
**17**	331.60
**18**	343.44
**19**	389.21
**20**	416.83
**21**	424.74
**22**	429.32
**23**	483.02
**24**	508.46
**25**	518.25
**26**	547.73
**27**	579.73
**28**	603.81
**29**	616.32
**30**	656.79
**31**	696.20
**32**	726.69
**33**	767.64
**34**	813.53
**35**	827.43
**36**	837.16
**37**	846.33
**38**	847.43
**39**	861.52
**40**	875.66
**41**	888.27
**42**	922.44
**43**	929.52
**44**	946.63
**45**	952.39
**46**	1042.57
**47**	1048.20
**48**	1060.84
**49**	1064.03
**50**	1073.78
**51**	1089.36
**52**	1217.04

**53**	1223.02
**54**	1254.98
**55**	1311.26
**56**	1355.59
**57**	1373.64
**58**	1408.10
**59**	1422.02
**60**	1433.50
**61**	1437.61
**62**	1442.99
**63**	1522.03
**64**	1537.75
**65**	1988.95
**66**	2289.94
**67**	3247.16
**68**	3252.38
**69**	3254.33
**70**	3257.92
**71**	3264.17
**72**	3265.03
**73**	3273.06
**74**	3275.08

7. Cartesian Coordinates (*Å*) and FSPE (*Eh*) of all relevant species

Table S18. Cartesian coordinates (Å) from the geometry optimization (D4-PBE/def2-TZVP, CPCM(MeCN)) of $[Cp\text{-}N]^-$ (*S* = 0, FSPE = -247.4115 Eh)

C	-1.2700	0.5780	-0.0000
C	-0.8322	-0.6982	0.0000
C	0.6339	-0.6985	0.0000
C	1.0723	0.5775	-0.0000
C	-0.0987	1.5197	0.0000
H	-2.2960	0.9270	-0.0000
H	-1.4378	-1.5999	0.0000
H	1.2392	-1.6005	0.0000
H	2.0984	0.9261	-0.0000
N	-0.0984	2.7824	0.0000

Table S19. Cartesian coordinates (Å) from the geometry optimization (D4-PBE/def2-TZVP, CPCM(MeCN)) of $[Cp\text{-}N]^-$ (*S* = 1, FSPE = -247.4164 Eh)

C	-1.2540	0.5991	-0.0000
C	-0.8092	-0.7159	0.0000
C	0.6109	-0.7162	0.0000
C	1.0563	0.5986	-0.0000
C	-0.0987	1.4685	0.0000
H	-2.2786	0.9478	-0.0000
H	-1.4399	-1.5981	0.0000
H	1.2413	-1.5986	0.0000
H	2.0810	0.9469	-0.0000
N	-0.0984	2.7815	0.0000

Table S20. Cartesian coordinates (Å) from the geometry optimization (D4-PBE/def2-TZVP, CPCM(MeCN)) of Ph-N (S = 0, FSPE = -286.0182 Eh)

C	-2.0760	1.3741	-0.0000
C	-2.1487	0.0007	0.0000
C	-0.9654	-0.7391	-0.0000
C	0.2910	-0.1317	-0.0000
C	0.3671	1.2414	0.0000
C	-0.8138	2.0563	0.0000
H	-2.9605	2.0009	-0.0000
H	-3.1032	-0.5128	0.0000
H	-1.0242	-1.8230	-0.0000
H	1.1847	-0.7449	-0.0000
H	1.3141	1.7692	0.0000
N	-0.7429	3.3677	-0.0000

Table S21. Cartesian coordinates (Å) from the geometry optimization (D4-PBE/def2-TZVP, CPCM(MeCN)) of Ph-N (S = 1, FSPE = -286.0763 Eh)

C	-2.0771	1.3815	0.0000
C	-2.1357	0.0079	-0.0000
C	-0.9661	-0.7526	-0.0000
C	0.2789	-0.1232	0.0000
C	0.3691	1.2487	0.0000
C	-0.8146	2.0415	0.0000
H	-2.9759	1.9856	0.0000
H	-3.0996	-0.4877	0.0000
H	-1.0248	-1.8343	-0.0000
H	1.1840	-0.7196	-0.0000
H	1.3277	1.7523	0.0000
N	-0.7435	3.3585	-0.0000

Table S22. Cartesian coordinates (Å) from the geometry optimization (D4-PBE/def2-TZVP, CPCM(MeCN)) of the ferrocenyl diazide ground state (S = 0, FSPE = -1977.3207 Eh)

Fe	0.0155	0.1144	0.3092
C	-0.6921	-1.3430	-0.9354
C	-1.0283	-1.6390	0.4259
C	0.2016	-1.7415	1.1583
C	1.2963	-1.4758	0.2639
C	0.7358	-1.2433	-1.0352
H	-1.4006	-1.1945	-1.7463
H	-2.0235	-1.7706	0.8422
H	2.3501	-1.4622	0.5308
H	1.2985	-1.0066	-1.9348
C	-0.8591	1.8575	-0.2983
C	-1.2070	1.5689	1.0619
C	0.0143	1.5083	1.8131
C	1.1190	1.7221	0.9171
C	0.5699	1.9520	-0.3873
N	0.0344	1.2947	3.2066
N	1.1202	1.2330	3.7841
N	2.0541	1.1575	4.4429
H	-1.5589	1.9588	-1.1238
H	-2.2050	1.4255	1.4675
H	2.1719	1.7154	1.1885
H	1.1434	2.1385	-1.2918
N	0.2390	-2.0783	2.5272
N	1.3272	-2.1178	3.1018
N	2.2650	-2.1898	3.7554

Table S23. Cartesian coordinates (Å) from the geometry optimization (D4-PBE/def2-TZVP, CPCM(MeCN)) of the ferrocenyl diazide ground state (S = 1, FSPE = -1977.2638 Eh)

Fe	0.0136	0.1296	0.2429
C	-0.6814	-1.4618	-0.9564
C	-1.0037	-1.7642	0.4179
C	0.2167	-1.9553	1.1204
C	1.2893	-1.5808	0.2474
C	0.7339	-1.3664	-1.0643
H	-1.4053	-1.3413	-1.7580
H	-1.9993	-1.9073	0.8304
H	2.3460	-1.5734	0.5053
H	1.3076	-1.1526	-1.9623
C	-0.8565	1.9826	-0.2812
C	-1.2017	1.6980	1.0906
C	-0.0049	1.7256	1.8567
C	1.0973	1.8264	0.9469
C	0.5603	2.0767	-0.3668
N	0.0136	1.4918	3.2373
N	1.0956	1.4086	3.8237
N	2.0230	1.3168	4.4927
H	-1.5677	2.1036	-1.0941
H	-2.2042	1.5684	1.4907
H	2.1501	1.8278	1.2205
H	1.1490	2.2827	-1.2569
N	0.2635	-2.2674	2.4848
N	1.3538	-2.3027	3.0608
N	2.2933	-2.3811	3.7145

Table S24. Cartesian coordinates (Å) from the geometry optimization (D4-PBE/def2-TZVP, CPCM(MeCN)) of the ferrocenyl diazide intermediate (S = 1, FSPE = -1977.2713 Eh)

Fe	-0.9862	0.0078	1.0110
C	-2.0013	-1.4725	0.0043
C	-1.8821	-1.8360	1.3860
C	-0.4860	-1.9503	1.6753
C	0.2639	-1.5753	0.5120
C	-0.6835	-1.3140	-0.5332
H	-2.9349	-1.3236	-0.5312
H	-2.6904	-2.0261	2.0866
H	1.3473	-1.5370	0.4326
H	-0.4351	-1.0241	-1.5507
C	-2.1039	1.6787	0.6617

C	-1.9828	1.4619	2.0689
C	-0.5809	1.5764	2.4325
C	0.1577	1.7048	1.1845
C	-0.7868	1.8298	0.1183
N	-0.1533	1.3642	3.6945
N	1.2540	1.3970	3.7616
N	1.9011	1.2181	4.7408
H	-3.0337	1.6889	0.0989
H	-2.7947	1.2929	2.7712
H	1.2384	1.7535	1.0958
H	-0.5423	1.9762	-0.9310
N	-0.0205	-2.2770	2.9533
N	1.1970	-2.2437	3.1700
N	2.2831	-2.2589	3.5167

Table S25. Cartesian coordinates (Å) from the geometry optimization (D4-PBE/def2-TZVP, CPCM(MeCN)) of the ferrocenyl diazide transition state (S = 1, FSPE = -1977.2251 Eh)

Fe	-1.0226	0.0060	0.9456
C	-1.9758	-1.5406	-0.0802
C	-1.9065	-1.8771	1.2994
C	-0.5387	-1.9590	1.6529
C	0.2444	-1.6087	0.5219
C	-0.6519	-1.3750	-0.5599
H	-2.8835	-1.4180	-0.6510
H	-2.7365	-2.0501	1.9676
H	1.3222	-1.5490	0.4865
H	-0.3629	-1.1080	-1.5650
C	-2.1051	1.6969	0.7313
C	-1.9648	1.4063	2.1132
C	-0.5697	1.5663	2.4980
C	0.1384	1.6749	1.2326
C	-0.8049	1.8600	0.1865
N	-0.1564	1.4188	3.7316
N	1.4873	1.4928	3.7103
N	2.1018	1.3320	4.6495
H	-3.0364	1.7386	0.1861
H	-2.7679	1.2037	2.8058
H	1.2106	1.7125	1.1270
H	-0.5663	2.0500	-0.8496
N	-0.1249	-2.2589	2.9515
N	1.0742	-2.1718	3.1968
N	2.1398	-2.1307	3.5417

Table S26. Cartesian coordinates (Å) from the geometry optimization (D4-PBE/def2-TZVP, CPCM(MeCN)) of the ferrocenylazide nitrene product (S = 0, FSPE = -1867.8373 Eh)

Fe	-0.9476	0.0538	1.1406
C	-1.9433	-1.3687	0.0519
C	-1.8833	-1.8189	1.4034
C	-0.4991	-1.9461	1.7386
C	0.3054	-1.5326	0.6181
C	-0.5972	-1.2021	-0.4344
H	-2.8514	-1.1683	-0.5100
H	-2.7158	-2.0349	2.0665
H	1.3911	-1.5095	0.5838
H	-0.3166	-0.8564	-1.4257
C	-2.1781	1.6814	0.6446
C	-2.0623	1.5040	2.0369
C	-0.6041	1.5041	2.3949
C	0.0995	1.7990	1.1004
C	-0.8634	1.8624	0.0753
N	-0.1253	0.6093	3.2168
H	-3.0952	1.6384	0.0600
H	-2.8616	1.2853	2.7402
H	1.1802	1.8373	0.9901
H	-0.6704	1.9755	-0.9900
N	-0.0834	-2.4287	2.9795
N	1.1084	-2.3147	3.2862
N	2.1683	-2.2829	3.7032

Table S27. Cartesian coordinates (Å) from the geometry optimization (D4-PBE/def2-TZVP, CPCM(MeCN)) of the ferrocenylazide nitrene product (S = 1, FSPE = -1867.8478 Eh)

Fe	-1.0056	-0.0089	0.9944
C	-1.9849	-1.4881	-0.0705
C	-1.9170	-1.8502	1.3151
C	-0.5332	-1.9469	1.6627
C	0.2593	-1.5752	0.5255
C	-0.6500	-1.3206	-0.5561
H	-2.8976	-1.3434	-0.6424
H	-2.7511	-2.0438	1.9840
H	1.3449	-1.5344	0.4865
H	-0.3662	-1.0295	-1.5642
C	-2.1263	1.6684	0.7083
C	-2.0021	1.3737	2.1000
C	-0.5790	1.5091	2.4852
C	0.1426	1.6533	1.2006
C	-0.8156	1.8397	0.1591
N	-0.0610	1.3581	3.6667
H	-3.0607	1.7144	0.1535
H	-2.8099	1.1871	2.8023
H	1.2239	1.7133	1.1120
H	-0.5839	2.0361	-0.8852
N	-0.1118	-2.2925	2.9573
N	1.0850	-2.1897	3.2393
N	2.1549	-2.1433	3.6369

Table S28. Cartesian coordinates (Å) from the geometry optimization (D4-PBE/def2-TZVP, CPCM(MeCN)) of the ferrocenylazide nitrene product (S = 0 open-shell, FSPE = -1867.8427 Eh)

Fe	-1.0049	-0.0070	1.0000
C	-1.9853	-1.4968	-0.0719
C	-1.9134	-1.8635	1.3129
C	-0.5298	-1.9693	1.6542
C	0.2587	-1.5856	0.5199
C	-0.6532	-1.3271	-0.5595
H	-2.9003	-1.3535	-0.6404
H	-2.7460	-2.0619	1.9823
H	1.3443	-1.5465	0.4774
H	-0.3700	-1.0352	-1.5674
C	-2.1239	1.6586	0.7042
C	-2.0051	1.3883	2.1023
C	-0.5931	1.6016	2.5134
C	0.1375	1.6679	1.2216
C	-0.8096	1.8298	0.1640
N	-0.0972	1.5370	3.6975
H	-3.0549	1.6864	0.1423
H	-2.8189	1.2101	2.8001
H	1.2189	1.7389	1.1402
H	-0.5666	2.0090	-0.8809
N	-0.1057	-2.3082	2.9480
N	1.0988	-2.2473	3.2135
N	2.1743	-2.2390	3.5972

Table S29. Cartesian coordinates (Å) from the geometry optimization (D4-PBE0/def2-TZVP, CPCM(MeCN)) of the ferrocenyl diazide ground state (S = 0, FSPE = -1977.2714 Eh)

Fe	0.0168	0.1128	0.3164
C	-0.6772	-1.3505	-0.9235
C	-1.0176	-1.6494	0.4226
C	0.1948	-1.7481	1.1560
C	1.2850	-1.4878	0.2773
C	0.7381	-1.2524	-1.0134
H	-1.3768	-1.1943	-1.7305
H	-2.0087	-1.7723	0.8319
H	2.3301	-1.4669	0.5485
H	1.3033	-1.0082	-1.9001
C	-0.8477	1.8606	-0.2806
C	-1.1973	1.5780	1.0669
C	0.0088	1.5128	1.8140
C	1.1065	1.7273	0.9326
C	0.5682	1.9527	-0.3634
N	0.0246	1.2885	3.2019
N	1.1135	1.2533	3.7534

N	2.0572	1.2040	4.3634
H	-1.5395	1.9543	-1.1039
H	-2.1895	1.4285	1.4646
H	2.1521	1.7121	1.2025
H	1.1425	2.1301	-1.2600
N	0.2226	-2.0771	2.5227
N	1.3131	-2.1250	3.0697
N	2.2587	-2.1988	3.6743

Table S30. Cartesian coordinates (Å) from the geometry optimization (D4-PBE0/def2-TZVP, CPCM(MeCN)) of the ferrocenyl diazide ground state (S = 1, FSPE = -1977.2407 Eh)

Fe	0.0140	0.1295	0.2515
C	-0.6666	-1.5050	-0.9473
C	-0.9928	-1.7940	0.4182
C	0.2124	-1.9492	1.1251
C	1.2807	-1.6127	0.2540
C	0.7342	-1.4175	-1.0514
H	-1.3821	-1.3889	-1.7474
H	-1.9831	-1.9276	0.8281
H	2.3294	-1.5966	0.5145
H	1.3057	-1.2068	-1.9428
C	-0.8427	2.0239	-0.2642
C	-1.1958	1.7338	1.0935
C	-0.0132	1.7258	1.8543
C	1.0841	1.8563	0.9648
C	0.5592	2.1189	-0.3387
N	-0.0035	1.4742	3.2333
N	1.0791	1.3801	3.7907
N	2.0148	1.2788	4.4082
H	-1.5427	2.1481	-1.0767
H	-2.1940	1.5996	1.4838
H	2.1289	1.8472	1.2411
H	1.1489	2.3221	-1.2198
N	0.2561	-2.2421	2.4954
N	1.3504	-2.2751	3.0375
N	2.3004	-2.3389	3.6379

Table S31. Cartesian coordinates (Å) from the geometry optimization (D4-PBE0/def2-TZVP, CPCM(MeCN)) of the ferrocenyl diazide intermediate (S = 1, FSPE = -1977.2316 Eh)

Fe	-1.0162	0.0123	0.9350
C	-1.9907	-1.5419	-0.0351
C	-1.8852	-1.8643	1.3453
C	-0.5081	-1.9480	1.6637
C	0.2448	-1.6096	0.5085
C	-0.6779	-1.3856	-0.5518
H	-2.9131	-1.4229	-0.5827
H	-2.6973	-2.0244	2.0384
H	1.3212	-1.5462	0.4456
H	-0.4141	-1.1284	-1.5664
C	-2.0857	1.7181	0.7128
C	-1.9428	1.4485	2.1001
C	-0.5531	1.5562	2.4611
C	0.1587	1.6895	1.2123
C	-0.7842	1.8677	0.1634
N	-0.1141	1.3679	3.7071
N	1.2615	1.4020	3.7536
N	1.9223	1.2502	4.7136
H	-3.0184	1.7646	0.1709
H	-2.7434	1.2516	2.7974
H	1.2318	1.7107	1.1065
H	-0.5454	2.0493	-0.8740
N	-0.0646	-2.2315	2.9526
N	1.1427	-2.1551	3.1663
N	2.2153	-2.1187	3.4860

Table S32. Cartesian coordinates (Å) from the geometry optimization (D4-PBE0/def2-TZVP, CPCM(MeCN)) of the ferrocenyl diazide transition state (S = 1, FSPE = -1977.2251 Eh)

Fe	-1.0226	0.0060	0.9456
C	-1.9758	-1.5406	-0.0802
C	-1.9065	-1.8771	1.2994
C	-0.5387	-1.9590	1.6529
C	0.2444	-1.6087	0.5219
C	-0.6519	-1.3750	-0.5599
H	-2.8835	-1.4180	-0.6510
H	-2.7365	-2.0501	1.9676
H	1.3222	-1.5490	0.4865
H	-0.3629	-1.1080	-1.5650
C	-2.1051	1.6969	0.7313
C	-1.9648	1.4063	2.1132
C	-0.5697	1.5663	2.4980
C	0.1384	1.6749	1.2326
C	-0.8049	1.8600	0.1865
N	-0.1564	1.4188	3.7316
N	1.4873	1.4928	3.7103
N	2.1018	1.3320	4.6495
H	-3.0364	1.7386	0.1861
H	-2.7679	1.2037	2.8058
H	1.2106	1.7125	1.1270
H	-0.5663	2.0500	-0.8496
N	-0.1249	-2.2589	2.9515
N	1.0742	-2.1718	3.1968
N	2.1398	-2.1307	3.5417

Table S33. Cartesian coordinates (Å) from the geometry optimization (D4-PBE0/def2-TZVP, CPCM(MeCN)) of the ferrocenylazide nitrene product (S = 0, FSPE = -1867.7947 Eh)

Fe	-0.9513	0.0339	1.1282
C	-1.9322	-1.3860	0.0382
C	-1.8774	-1.8113	1.3898
C	-0.5083	-1.9358	1.7334
C	0.2898	-1.5394	0.6201
C	-0.6020	-1.2269	-0.4356
H	-2.8318	-1.1853	-0.5234
H	-2.7087	-2.0126	2.0473
H	1.3683	-1.5056	0.5894
H	-0.3179	-0.8803	-1.4177
C	-2.1632	1.6848	0.6698
C	-2.0457	1.4903	2.0406
C	-0.5966	1.4763	2.3926
C	0.0980	1.7799	1.1082
C	-0.8556	1.8623	0.1012
N	-0.1191	0.6153	3.2254
H	-3.0755	1.6466	0.0914
H	-2.8394	1.2659	2.7375
H	1.1713	1.8068	0.9921
H	-0.6651	1.9749	-0.9570
N	-0.0985	-2.4117	2.9765
N	1.0823	-2.2640	3.2709
N	2.1333	-2.1914	3.6520

Table S34. Cartesian coordinates (Å) from the geometry optimization (D4-PBE0/def2-TZVP, CPCM(MeCN)) of the ferrocenylazide nitrene product (S = 1, FSPE = -1867.8176 Eh)

Fe	-1.0075	-0.0032	0.9875
C	-1.9727	-1.5109	-0.0767
C	-1.9110	-1.8609	1.3010
C	-0.5434	-1.9471	1.6566
C	0.2465	-1.5966	0.5296
C	-0.6489	-1.3471	-0.5504
H	-2.8770	-1.3700	-0.6490
H	-2.7435	-2.0429	1.9636
H	1.3250	-1.5499	0.4955
H	-0.3594	-1.0625	-1.5506
C	-2.1115	1.6780	0.7294
C	-1.9860	1.3818	2.1064
C	-0.5766	1.5218	2.4884

C	0.1374	1.6601	1.2145
C	-0.8115	1.8477	0.1835
N	-0.0608	1.3568	3.6595
H	-3.0390	1.7145	0.1772
H	-2.7885	1.1883	2.8015
H	1.2113	1.7126	1.1214
H	-0.5813	2.0341	-0.8552
N	-0.1296	-2.2799	2.9536
N	1.0601	-2.1557	3.2143
N	2.1225	-2.0825	3.5693
